# Should I stay, or should I go: Anthropogenic noises disrupt early recruitment of subarctic invertebrates

**DOI:** 10.1002/eap.70119

**Published:** 2025-11-10

**Authors:** Nathália Byrro Gauthier, Thomas Uboldi, Frédéric Olivier, Réjean Tremblay, Laurent Chauvaud, Delphine Mathias, Pascal Lazure, Antoine Frémont, Tarik Meziane, Sylvain Chauvaud, Gesche Winkler

**Affiliations:** ^1^ Institut des Sciences de la Mer (ISMER), Université du Québec à Rimouski (UQAR) Rimouski Québec Canada; ^2^ Laboratoire des Sciences de l'Environnement Marin (UMR 6539 LEMAR) UBO, CNRS, IRD, Institut Français de Recherche pour l'Exploitation de la Mer (IFREMER), Institut Universitaire Européen de la Mer (UAR 3113 IUEM) Plouzané France; ^3^ Laboratoire de ‘Biologie des Organismes et Écosystèmes Aquatiques’ (UMR 8067 BOREA) Muséum National d'Histoire Naturelle (MNHN), Sorbonne Université (SU), Université des Antilles (UA), Centre National de la Recherche Scientifique (CNRS), Institut de Recherche pour le Développement (IRD 207) Paris France; ^4^ Université de Bretagne Occidentale (UBO), CNRS, IRD, IUEM Plouzané France; ^5^ Société d'Observation Multimodale de l'Environnement (SOMME) Brest France; ^6^ Laboratoire d'Océanographie Physique et Spatiale (UMR 6523 LOPS) UBO, IRD, IFREMER, CNRS, IUEM Plouzané France; ^7^ Laboratoire Interdisciplinaire pour la Sociologie Économique (UMR 3320 LISE) Conservatoire National des Arts et Métiers (CNAM), CNRS Paris France; ^8^ SAFIR, Sorbonne University Abu Dhabi Abu Dhabi United Arab Emirates; ^9^ Télédétection en Biologie Marine et Environnement (TBM Environnement) Auray France

**Keywords:** anthropophony, bivalves, gastropods, marine noise pollution, noise effects, population dynamics

## Abstract

Coastal subarctic systems are inhabited by bivalve and gastropods, which due to their lifecycle and longevity are reliable indicators of ecological alterations in the environment. Recent laboratory studies have shown that young life stages of invertebrates perceive natural sounds, and their settlement, behavior, and fitness could be altered by anthropogenic noise. Through a field study conducted on two sites differing by their noise pollution level (pristine [PS] or anthropized [AS]), we tested whether the distances (from 25 to 890 m) of anthropogenic noises might affect the diversity and early recruitment of multiple species in pristine and anthropized sites using artificial collectors moored on transects. Overall, environmental conditions (except sound levels) were homogeneous through the transects. The acoustic scenario differed between the PS (vessel noise, 132–138 dB re 1 μPa^2^ s) and AS (mix of pile driving and vessel noise, >140 dB re 1 μPa^2^ s) sites, with the AS site experiencing a higher level of sound exposure than the PS site. Species richness fluctuated with distance from the noise, but only in the anthropized site. Regarding species diversity and evenness, they varied with distance and month at both sites, displaying a clear negative effect of anthropogenic noises and shifting species composition. Specific early recruitment responses were observed for each species to anthropogenic noise, but with a different pattern for each site due to variations in sound pressure and exposure levels. The findings of our field study document, for the first time, that controlled anthropogenic noise emission leads to ecological shifts in community structure and population metrics of benthopelagic marine invertebrate species. To avoid disruptions in community structure and recruitment, we recommend that a noise threshold level for invertebrates should be below 140 dB re 1 μPa^2^ s.

## INTRODUCTION

Subarctic ecosystems are dynamic and fragile environments, characterized by a remarkable diversity of marine organisms, including the ubiquitous marine invertebrates (Dunbar, 1954). These organisms are essential components of coastal ecosystems and serve as reliable indicators of environmental disturbances (Baden et al., 2021; Seed et al., 2000). Over the years, the subarctic region has been increasingly enduring multiple anthropogenic stressors. These include ocean warming and acidification, chemical pollution (see details on Halpern et al., 2019; Svavarsson et al., 2021), and anthropogenic noise—recently recognized as a distinct and pervasive form of pollution (Duarte et al., 2021; Merchant et al., 2022; Slabbekoorn et al., 2010). Collectively, these pressures jeopardize the health and stability of the ecosystem at risk (Halpern et al., 2019). Therefore, monitoring subarctic communities through metrics such as species richness, diversity, and evenness became vital. These ecological indicators offer insights into recruitment patterns, help to predict responses to anthropogenic disturbance, and support the development of sustainable mitigation and management strategies (Austen et al., 2002; Campagne et al., 2023; Dreujou et al., 2020). Among emerging stressors, anthropogenic noise (such as vessel noise, pile driving, drilling, and related activities) has gained increasing attention. Some countries have incorporated such noise into marine directives or are working toward guidelines to address its ecological implications to prevent harmful effects (Breeze et al., 2022; Merchant et al., 2022). The frequency, intensity, and temporal characteristics of anthropogenic noises turn them into pervasive pollutants, capable of generating ecological effects across both terrestrial and marine ecosystems (Jerem & Mathews, 2020; Kunc et al., 2016; Sordello et al., 2020). Despite growing research into its effects on wildlife, birds and marine mammals remain the most intensively studied groups, with invertebrates and reptiles receiving the least attention (Jerem & Mathews, 2020; Sordello et al., 2020). Noise has been shown to alter bird diversity (Perillo et al., 2017; Proppe et al., 2013) and amphibian species composition (Trowbridge & Litzgus, 2022). However, the ecological effects of anthropogenic noise on marine benthic invertebrates remain understudied, despite emerging evidence that noise promotes behavioral and fitness effects (Gigot et al., 2024; Jolivet et al., 2016; Olivier et al., 2023; Stocks et al., 2012; Veillard et al., 2025; Wilkens et al., 2012).

One particularly sensitive phase in the life cycle of benthic invertebrates, like bivalves and gastropods, is the transition from planktonic larvae to benthic sessile or slow‐moving juveniles. These stages are connected by a milestone event filled with exploratory behavior and cue detection, known as settlement (Hadfield & Paul, 2001; Thorson, 1950). Numerous studies have demonstrated that larvae evaluate a range of cues when selecting or rejecting settlement sites, including hydrodynamics (Butman, 1987; Fuchs et al., 2018; Pernet et al., 2003), substrate characteristics (Bayne, 1965; Fraschetti et al., 2003), biofilm composition (Hadfield & Paul, 2001; Toupoint, Mohit, et al., 2012), the presence of conspecifics or predators (Beal et al., 2020; Hadfield & Paul, 2001; Morello & Yund, 2016), trophic signals (Androuin et al., 2022; Toupoint, Gilmore‐Solomon, et al., 2012), and, more recently, underwater soundscapes (Lillis et al., 2013, 2015; Williams et al., 2022). Some of these acoustic cues are anthropogenic in origin (i.e., maritime traffic, pile driving or drilling operations), and recent research suggests that larvae can perceive and respond to them. Laboratory studies have shown that larvae may respond or not to the combination of trophic cues with anthropogenic noise (Gigot, Olivier, et al., 2023; Gigot, Tremblay, et al., 2023; Jolivet et al., 2016). If a suitable substrate and environmental conditions are found, larvae settle and metamorphose into a benthic form (Hunt & Scheibling, 1997). However, in the absence of suitable cues, settlement–metamorphosis of species may be delayed. This delay not only provides larvae with another opportunity to locate a suitable settlement site but also increases their vulnerability to environmental stressors (Bayne, 1965; Lagarde et al., 2018; Martel et al., 2014; Pechenik, 1990). Post‐metamorphic stages are also vulnerable, but in this case, substantial mortality is often driven by predation (reviewed by Beal et al., 2020; Gosselin & Qian, 1997; O'Connor et al., 2008). In some species, this first attachment is not permanent, and post‐metamorphic stages may passively resuspend or actively respond to environmental stimuli (i.e., hydrodynamics, trophic environment, pollution), resulting in secondary migration and new spatial distribution (Forêt et al., 2018; Günther, 1992; Martel & Chia, 1991). Consequently, recruitment is governed not only by larval availability (supply‐side theory) but also by larval behavior during settlement, physiological state, and post‐settlement survival and migration, all modulated by a complex interplay of biotic and abiotic factors (Hunt & Scheibling, 1997; Keough & Downes, 1982; Leal et al., 2022; Martel & Chia, 1991).

Shipping noise is increasingly recognized as a disruptor of natural soundscapes (Duarte et al., 2021) with potential consequences for population processes and community structure (Kunc et al., 2016; Solé et al., 2023). To progress in this context, field experiments are required, as they offer ecological insights by incorporating seasonal variation, species interactions, and simultaneous environmental stressors (Spicer, 2014). This limitation is compounded by the lack of in situ data on the distances from the source at which noise begins to impair invertebrate functions, making it difficult to define biologically relevant thresholds (Hawkins & Popper, 2017). In contrast, regulatory thresholds for sound pressure and exposure levels have been more thoroughly established for marine mammals, and to a lesser extent, for fish. Similarly, regulatory efforts across other taxa and countries remain uneven compared to marine mammals (see Bonnel et al., 2022; Breeze et al., 2022; Merchant et al., 2022; NFMS, 2018; Popper et al., 2014 for an overview of the topic).

Understanding how vessel noise affects organisms at specific distances from its source is especially important for invertebrate communities and foundation species inhabiting both nearly pristine and more anthropized environments, where differing acoustic soundscapes may lead to distinct impacts (Halliday et al., 2021; NRC, 2003). To address this, we selected experimental sites with contrasting acoustic profiles to assess whether a gradient of vessel noise produces different ecological responses at each site. We deployed artificial collectors at three distances (D1, D2, and D3) from a commercial vessel noise source along transects at two sites with different levels of maritime traffic: Miquelon (pristine) and Saint‐Pierre (anthropized). This setup exposed collectors to a gradient of vessel noise intensities: 137 dB at D1, 120 dB at D2, and 106 dB re 1 μPa at D3. Therefore, the main objective of this study was to assess the effect of distances from such anthropogenic noise—measured as sound intensity—on the diversity and early recruitment of multiple benthic species in a field setting. We hypothesized that as the distance between the re‐emitted vessel noise and collectors decreases, the diversity and early recruitment of subarctic invertebrates would also decline. To test this hypothesis, we first estimated the number of taxa, diversity, and evenness in the collectors, using indices such as species richness (*S*), Shannon–Wiener (*H*′), and Pielou's Evenness (*J*′). We then examined the abundance (potential settlers, post‐larvae, juveniles) from different invertebrate taxa on collectors placed at increasing distances from vessel noise sources over 4 months in the field.

## MATERIALS AND METHODS

### Study sites

The study was carried out in Saint‐Pierre and Miquelon (SPM), a French archipelago in the Northwest Atlantic Ocean, which is surrounded by the Grand Banks, the Gulf of Saint‐Lawrence, and the Scotian Shelf (Dubois, 2010; IEDOM, 2022, p. 20, Figure 1). SPM lies at the convergence of the warm Gulf Stream and the cold Labrador Current, creating a subarctic environment with a strong seasonal thermocline. These currents (Labrador and Scotian Shelf) are generally weak during spring, but they intensify during fall and winter. SPM experiences large near‐bottom diurnal temperature amplitude, with low and high stratification periods in early July and late August, respectively. Tides are mostly semidiurnal of relatively low range (~1.3 m) (Lazure et al., 2018). The trophic environment is mostly oligotrophic, with spring and occasional microalgal blooms (April, September/October). Seston quality, assessed by fatty acid methods, is influenced by stratification conditions, which might potentially impact nutrient exchange between surface and subsurface waters (Bridier et al., 2021); however, this thermal‐trophic variability is lower in shallow waters (<20 m). Additionally, the relatively flat shallow seafloor (Lazure et al., 2018) makes SPM ideal for in situ acoustic playback experiments (Bonnel et al., 2022), as sound propagation in shallow waters depends heavily on depth and physical properties of the seafloor (NRC, 2003). Together, these features make SPM a suitable site to conduct in situ experiments that assess the impact of noise on marine organisms. To minimize variability in the physical and trophic conditions—to avoid confounding effects from sound propagation—we selected two sites: a pristine site (Miquelon, pristine, PS) with no industrial marine traffic, and an anthropized site (Saint‐Pierre, anthropized, AS) near an industrial harbor. For both sites, vessel activity was obtained using the Automatic Identification System (AIS) from MarineTraffic Professional Plus software (www.marinetraffic.com). At the PS site, only a small ferry and a few recreational boats were detected, whereas 103 vessel trajectories were recorded at the AS between May 12 and 29, 2021.

**FIGURE 1:**
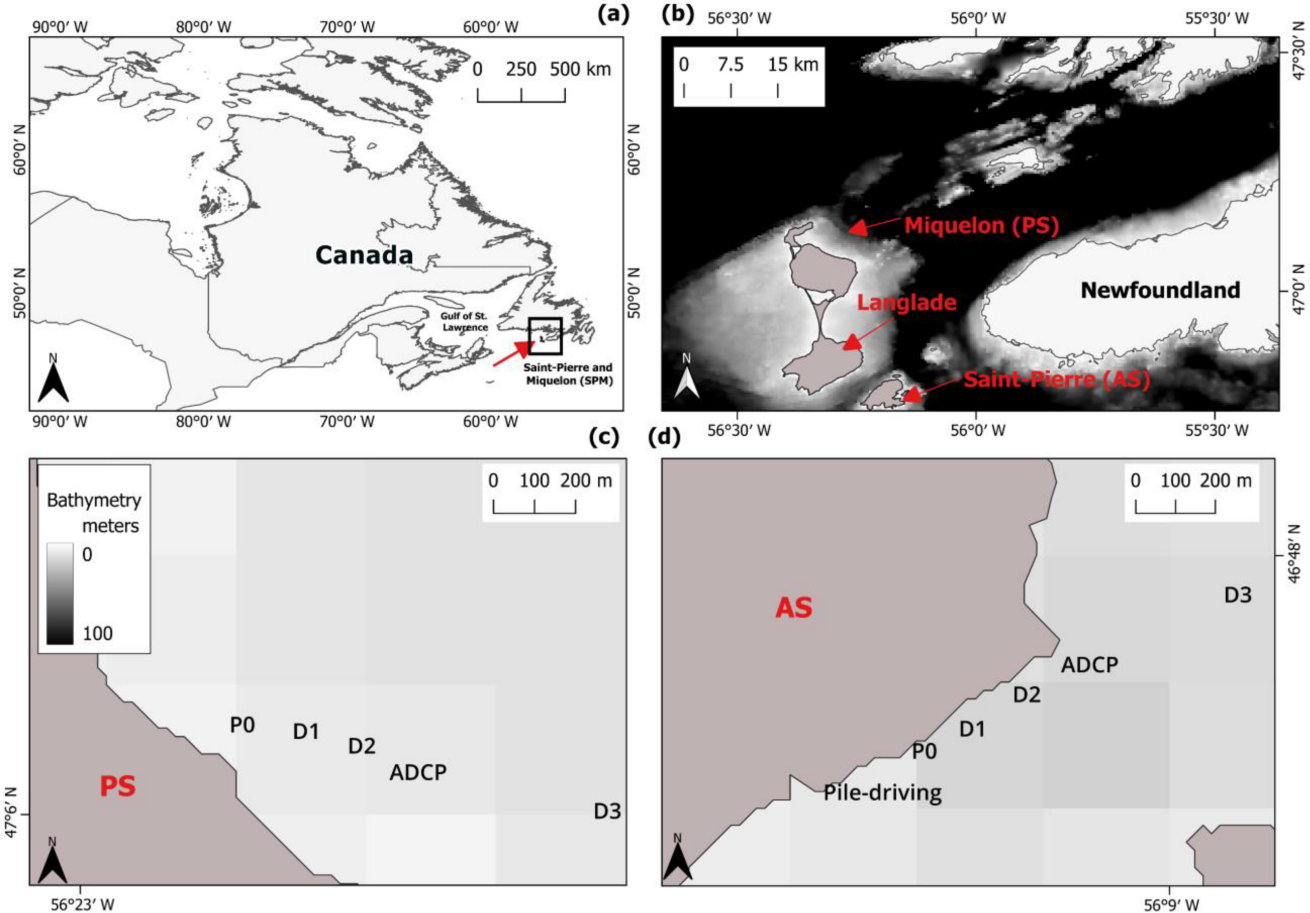
Set of maps of our study area: (a, b) Location of Saint‐Pierre and Miquelon's archipelago, where each site is referred as pristine (PS) or anthropized (AS) due to the level of anthropogenic noise at the islands of Miquelon and Saint‐Pierre, respectively. (c, d) Bathymetric maps containing sites, and stations from the sound (D1, D2, and D3) on transects exposed to an anthropogenic noise source (P0) at pristine site (PS) and anthropized site (AS), which had pile driving operations occurring at the same time of sampling. Bathymetry data were retrieved from General Bathymetric Chart of the Oceans (GEBCO). Bathymetric maps are composed using grayscale where white represents 0–1 m, 2–30 m light to dark gray, and values >100 m are black. ADCP, acoustic Doppler current profilers.

### Vessel noise recordings and soundtrack compilation

Acoustic recordings were collected from the AS site (46°46′44.90″ N, −56°10′38.28″ W) from November 2020 to April 2021 using a calibrated hydrophone (Aural‐M2, Multi‐Électronique, Rimouski, Canada) moored close to the seabed at the Saint‐Pierre harbor. Recordings made at the AS site contain noise from the “*Nolhan Ava*,” a 120‐m commercial ship that provides cargo services to SPM. The spectral composition and source sound level were also determined using the software MATLAB (2021a). From these recordings, a 1‐h and 20‐min maritime traffic soundtrack was created, alternating between vessel noise (11 min, then 8.5 min) and silent segments (39 min, then 21.5 min), as in Veillard et al. (2025). This vessel noise soundtrack was played and looped during the experimental period from July to October 2021 at each site (PS and AS) using underwater speakers (Lubell Labs VC2C, Columbus, OH, USA), which emitted approximately a root mean square sound pressure level (SPL_rms_) of 167 dB re 1 μPa of vessel noise at 0 m (Table 1).

**TABLE 1:** Location and sampling points, distances from the speaker (in meters), point coordinates (degrees, minutes, seconds), depth (in meters), and levels of sound received (decibels relative to a reference pressure of one micropascal) at each distance sampled in the Pristine (PS, Miquelon) and Anthropized (AS, Saint‐Pierre) sites.

Sites and sampling distances	Distance from speaker (m)	Received level of sound (dB re 1 μPa)	Depth (m)	Coordinates	
				Latitude	Longitude
PS					
P0	0	167		47°06′08.39″ N	56°22′28.62″ W
D1	25	137	8	47°06′08.22″ N	56°22′27.59″ W
D2	175	120	8	47°06′06.66″ N	56°22′20.70″ W
D3	890	106	8	47°05′59.88″ N	56°21′48.53″ W
ADCP				47°06′04.38″ N	56°22′12.54″ W
AS					
P0	0	154		46°47′39.00″ N	56°09′29.58″ W
D1	30	153	15	46°47′38.46″ N	56°09′28.44″ W
D2	144	150	20	46°47′41.28″ N	56°09′23.64″ W
D3	848	143	20	46°47′52.73″ N	56°08′54.96″ W
ADCP				46°47′45.06″ N	56°09′16.02″ W

Abbreviation: ADCP, acoustic Doppler current profilers.

### Transmission loss calculations and acoustic features of pristine and anthropized sites

Transmission loss calculations using the formula TL = 20log*R* (where *R* is the range in meters) were made to estimate sound intensities received at each distance on each transect (Table 1). Based on the transmission loss calculation, these distances received different levels of SPL_rms_ ranging from 106 to 137 dB re 1 μPa in both sites (Table 1). The characterization of ambient noise was conducted for the two sites (Appendix S1: Figure S1). Due to the low levels of anthropogenic activity in the PS site, underwater acoustic recorders (RTSYS‐RESEA 320, Caudan, France; Hydrophone Colmar GP1516M, Sensitivity −172 dB re 1 V/μPa @ 5 kHz) were deployed 2 days in August, 2 days in September, and 1 day in October. An AURAL hydrophone was deployed continuously a month before starting the trial (June) and during the entire experimental period (July–October 2021) in the AS site. The sound exposure level per minute (SEL_1min_, total sound energy of one minute event in decibels relative to a reference pressure of one micropascal squared per second) was also calculated, and acoustic metrics were analyzed according to Bonnel et al. (2022). Therefore, we characterized sound levels at each site using SPL_rms_ and SEL_1min_.

### In situ experimental design

This study was carried out from the end of June to mid‐October 2021 in SPM. Underwater speakers (Lubell Labs VC2C, 50 Hz–1.5 kHz) protected by a metal frame were suspended to a depth of 8 m at both sites oriented offshore. Both transects were exposed to a replayed soundtrack of vessel sound. A set of collectors made of 2‐mm mesh bags, each filled with four inverted Netron sections (40 × 80 cm with a mesh size of 5 mm, area of 0.320 m^2^), were moored at three stations from the sound emission: D1 (close, 137 dB re 1 μPa at 25–30 m), D2 (intermediate, 120 dB re 1 μPa at 144–175 m), and D3 (far, 106 dB re 1 μPa at 848–890 m) (Appendix S1: Figure S1). On each mooring, four artificial collectors were installed close to the bottom and were kept in place by subsurface buoys, as detailed in Cyr et al. (2007). A total of 96 collectors were assessed during the experimental period (4 replicates × 3 distances along the transect × 4 months—July, August, September, October × 2 sites—PS and AS). The sanitary restrictions related to COVID‐19 downscaled our original design since it was unfeasible to have control transects at each site without having enough personnel to carry out the manipulation. For these reasons, the collectors at station D3 (far distance) in the PS site were considered as control since the sound reaching these collectors had similar levels of ambient sound (SPL_rms_ = 106 dB re 1 μPa) than levels documented in the literature (Halliday et al., 2021; Mathias et al., 2016).

### Monitoring environmental conditions of an in situ recruitment

Temperature and currents were monitored throughout the entire experimental period at each site. Temperature was measured by probes (HOBO Pendant Temperature/Light 64 K, contained within waterproof casings), while current velocity was determined using two acoustic Doppler current profilers (ADCP Teledyne RD Instruments 600 and 1200 kHz models, refer to Table 1 for position on the transect at each site). To monitor the distribution pattern of total particulate matter (TPM, hereafter referred to as seston), seawater samples (*n* = 2, 5 L each) were collected monthly at each distance (D1–D3) of both transects at each site at the mooring depths using Niskin bottles and stored in opaque vials. Two bottles per sampling were used as replicates for determining seston quantity (TPM, in milligrams per liter of dry weight) and quality as mass of total fatty acids (MTFA, fatty acid composition). After being collected, samples were filtered on pre‐weighed and pre‐combusted 47 mm GF/F filters and stored at −40°C for a maximum of 4 months, then transferred to −80°C until further analysis as described in Toupoint, Gilmore‐Solomon, et al. (2012). Additionally, at each sampling event, another 3 × 4.5 mL of pre‐filtered seawater (over a 40‐μm mesh) were fixed with 20 μL glutaraldehyde (0.1% final v/v) and kept at −40°C for determination of bacteria, pico‐ and nanoplankton using flow cytometry. Samples were stored at −40°C for up to 4 months, then transferred to −80°C until analyses.

### Response to anthropogenic noise: Community dynamics and early recruitment

During this experiment, the first collectors were moored at the end of June and retrieved by divers at the end of July 2021 and transported to the shore in individual bags to avoid spat losses. To retrieve all organisms inside, each collector (replicate) was placed in a 100‐μm plankton net and cleaned using a low‐pressure seawater jet. The material accumulated in the net cup was filtered on a 100‐μm mesh and transferred to labeled Ziplock bags and stored at −40°C for further analysis. In the laboratory, all species were sorted, counted, and identified to family, genus, or species level, when possible, under a stereomicroscope. The identification of species and families was made using the following taxonomic keys: Aucoin et al. (2004), Hayward and Ryland (2017), and Lutz et al. (2018).

### Laboratory procedures

#### Characterization of trophic environment: Fatty acid extraction and trophic markers

Several sources of organic matter contribute to feeding benthic species (diatoms, dinoflagellates, bacteria, macroalgae). Fatty acids (FA) are useful trophic markers to monitor the seston quality being delivered to recruits in the benthic realm (Bridier et al., 2021; Kelly & Scheibling, 2012; Meziane et al., 2007). Given the role of seston, particularly its quality of polyunsaturated fatty acids (PUFA) and essential fatty acids (EFA), in mussel settlement (Martel et al., 2014; Toupoint, Gilmore‐Solomon, et al., 2012), we analyzed the FA profile of seston samples collected at the two study sites. To process seston filters collected over the sampling period, we adopted biochemical techniques such as FA extraction and quantification. Detailed information on the methodology for extracting FA, FA composition, and trophic markers is available in Appendix S1: Section S1.

#### Flow cytometry analyses to determine food sources

Various food sources (e.g., picoplankton, nanoplankton, bacterial concentration) have been identified as mediators of bivalve settlement and early recruitment (Androuin et al., 2022; Lagarde et al., 2018; Toupoint, Gilmore‐Solomon, et al., 2012; Veillard et al., 2023). In conditions where planktonic sources are limited, biofilm cues (its composition) may play an important role in triggering settlement (Toupoint, Mohit, et al., 2012). To characterize the concentration of food sources available at our study sites, seston samples were analyzed using an Epics Altra flow cytometer (Beckman Coulter Inc., Fullerton, CA, USA). Fluorescently labeled cells were identified and quantified into different categories, including pico‐ (0.2–2 μm) and nano‐ (2–20 μm) eukaryotes, cyanobacteria, as well as heterotrophic bacteria, following the methods outlined by Belzile et al. (2008) and Tremblay et al. (2009), as detailed in Toupoint, Gilmore‐Solomon, et al. (2012).

#### Identification and abundance of benthic invertebrates

Samples were sieved over decreasing mesh sizes (1.7 mm, 500 and 150 μm) using seawater to distinguish primary settlers (>150 μm), from secondary settlers (>500 μm) and from larger juveniles (>1.7 mm). Fractions were sorted in a basin (>1.7 mm) and in a Dollfus counting chamber (>500 and >150 μm) under a Leica microscope (M125, Germany). Due to the high abundance of individuals, only a quarter of the samples from September and October were analyzed, and splits were prepared by using a Folsom splitter. Sub‐sampling adjustments were made for high abundance species such as *Hiatella arctica* and Mytilidae (25 squares spread out in eight square intervals). For low abundance species, individuals were counted in all 200 squares in the chamber. Even though individuals from *Mytilus* spp. and *Modiolus* spp. were identified to species level in larger fractions, the smallest fraction (>150 μm) was considered as Mytilidae. Since only a few taxa could not be identified to the species or family level, they were named after the group followed by a species number (i.e., Bivalve sp.1, Gastropod sp.1).

#### Community and species selection

The community composition was described using percentages of abundance. The community diversity was evaluated using different indices such as species number (Richness, *S*), the diversity (Shannon–Wiener Index, *H*′), and equitability (Pielou's evenness index, *J*′) of assemblages retrieved at different distances at the PS and AS sites. These indices were estimated without *H. arctica* because this species was overwhelmingly dominant in our collectors at both sites. To compare the effect of sound level exposure on species recruits, we chose five taxa based on the following conditions: (1) species or family that have been used as suitable environmental indicators for decades such as *H. arctica*, Mytilidae species, and *Placopecten magellanicus* (Cyr et al., 2007; Forêt et al., 2018; Garcia et al., 2003; Veillard et al., 2023); (2) foundation species such as *Modiolus* sp. and *Mytilus* spp. (= Mytilidae) (Baden et al., 2021; Seed et al., 2000); (3) important commercial and ecological bivalve and gastropod species such as *P. magellanicus* (Aucoin et al., 2004; Cyr et al., 2007; Tremblay et al., 2020) and *Lacuna* spp. (Martel & Chia, 1991; Martel & Diefenbach, 1993); and (4) taxa such as *Skenea* sp. that were present (>40 individuals month^−1^) at both sites.

### Data analysis

At the PS site, due to data loss, temperature differences were only assessed between D1 and D2 using the nonparametric Mann–Whitney test. At the AS site, variations in temperature were assessed between stations (D1, D2, D3). Data were transformed using the square root function, and residuals were tested for normality and homogeneity with the Shapiro–Wilk and Levene tests before conducting one‐way ANOVA.

Achieving a normal distribution of biological and trophic data is challenging when there is a wide variation in replicates and missing data at some sampling points (Anderson, 2001). Because of this, permutational multivariate ANOVA (PERMANOVA) was used with PRIMER‐E 7.0 (9999 permutations) to detect differences in trophic and biological data among distances at each site. Seston quantity (TPM) and quality (MTFA, fatty acid composition, and trophic markers, *n* = 6 or 8 depending on site) as well as food sources (heterotrophic bacteria, pico‐ and nanocyanobacteria, and pico‐ and nanoeukaryotes, *n* = 3 or 4 depending on site) were compared between stations at each site (3 levels—D1, D2, and D3) using one‐way PERMANOVA. TPM and MTFA matrices were individually constructed using the Euclidean distance, while food source data were ranked, due to different concentration levels, and then converted together into an Euclidean distance matrix. A Bray–Curtis dissimilarity matrix was calculated based on trophic marker data comprising bacteria (∑ai15:0, iso15:0, 18:1ω7), degraded organic matter (∑14:0, 16:0, 18:0), diatoms (∑16:1ω7, 20:5ω3), dinoflagellates (∑22:6ω3), green (∑18:2ω6, 18:3ω6), and brown macroalgae (∑18:1ω9). Significant PERMANOVA results (*p* < 0.05) were followed by pairwise tests, and similarity percentage (SIMPER) analyses were conducted on untransformed data to identify dissimilarities in all metrics within each distance from the underwater speaker (D1, D2, and D3). We estimated species abundances (in number of individuals per collector) from our biological data and then expressed each species/taxa in a Bray–Curtis similarity matrix. Diversity indices were calculated from this biological dataset using the function DIVERSE on PRIMER 7.0, and each calculated index was converted into an Euclidean distance matrix. These distance matrices were analyzed each using a two‐factor PERMANOVA to examine the effect of distance from the noise (Di, 3 levels—D1, D2, and D3), month (Mo, 4 levels—July, August, September, and October), and their interaction (Di × Mo) on the early recruitment, species richness, diversity, and evenness at each site. If results were significant (*p* < 0.05), pairwise comparisons were performed coupled to a Monte Carlo simulation test to validate the probability of our results when a limited number of permutations were executed. We also conducted SIMPER analyses to identify the species that most contributed (in percentage) to dissimilarities between collector assemblages at each distance and month. All statistical tests used a 0.05 significance level, performed with PRIMER 7.0 and GraphPad Prism 10.5. Plots were generated using GraphPad Prism 10.5 and MATLAB2021a.

## RESULTS

### Physical environment

**FIGURE 2:**
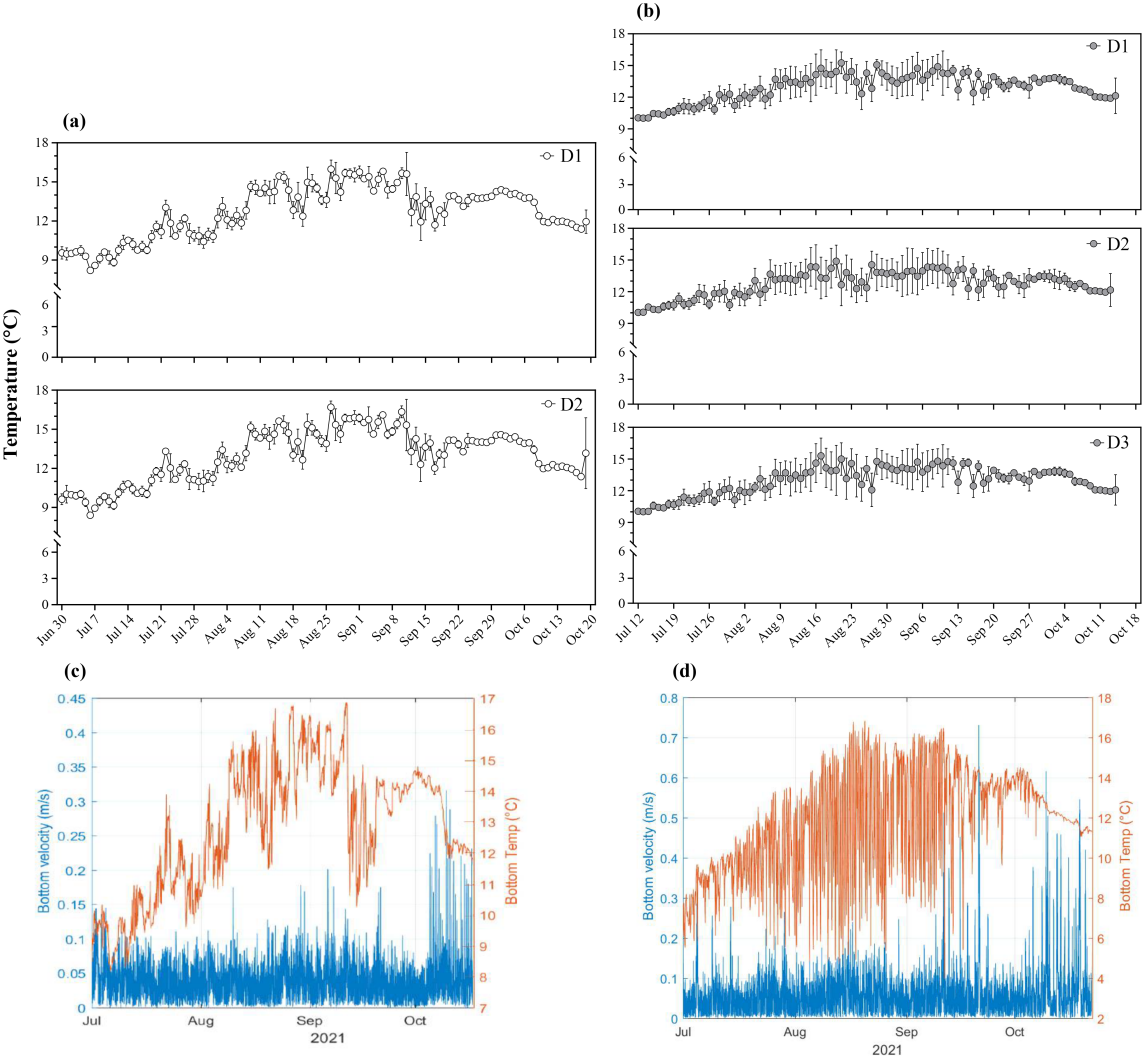
Temperature (in degrees Celsius) and current profiles (velocity, in meters per second) on each transect at pristine (PS, a, c) and anthropized (AS, b, d) sites during the sampling period from July to October 2021.

Temperatures (mean ± SD) in PS were uniform across distances (13.1 ± 0.5°C, *U* = 6934, *p* = 0.93), with seasonal fluctuations ranging from 8.5 to 17.0°C (Figure 2a). A 5°C drop and a 3°C rise in mid‐ and late September (Figure 2a), likely due to Hurricane Larry (Brown, 2021), resulted from a vertical mixing of the water column and heat loss from strong winds. In October, temperatures dropped from 15 to 11.5°C. At the AS site, temperatures were comparable along the transect (12.1 ± 1.5°C, ANOVA, df = 3, *F* = 0.41, *p* = 0.74), fluctuating seasonally between 5.5 (early July) and 16–17°C (mid‐August). Greater semidiurnal and diurnal oscillations (7–10°C) from mid‐August to mid‐September (Figure 2b) were driven by internal waves caused by barotropic tides, facilitated by stronger stratification at AS. The barotropic tide generates internal waves that cause the cold‐water masses on the bottom to oscillate in the cross‐shore direction at the tidal frequency (Lazure et al., 2018). These oscillations weakened after the September 11 storm due to increased vertical mixing. The seasonal temperature gradient in the PS site (2–4°C) was over two times lower than in the AS site (7–10°C), possibly due to an intense effect of internal waves in the AS site (Lazure et al., 2018).

Currents at PS ranged from 0 to 0.4 m s^−1^, with greater variability at the beginning of October (Figure 2c). Bottom and surface currents were weak and predominantly north–south and north‐west, respectively. Residual currents followed the bottom current pattern and moved toward north. Tides were semidiurnal to mixed semidiurnal, ranging from 1.3 to 2.0 m (Appendix S1: Figure S2a,b). Bottom pressure sensors recorded a tidal cycle on September 11, whose range rose from 1.7 m for the preceding and following tides to 2.5 m, likely caused by the passage of a storm‐triggered continental shelf wave over Newfoundland, as described by Brown (2021).

At the AS site, current speed ranged from 0 to 0.3 m s^−1^ from July to early September (Figure 2c), rising to 0.5–0.7 m s^−1^, mixing the water column and shifting the prior temperature amplitude. Bottom and surface currents were predominantly east‐northeast, with residual currents strengthening slightly in autumn and moving northward. Tidal patterns were similar to those at PS, varying from 1.3 to 2.0 m (Appendix S1: Figure S2c,d).

### Trophic environment

Seston quantity (TPM) and quality (MTFA, fatty acid composition, food sources, trophic markers) (mean ± SE) available for recruits were homogeneous along both transects, as shown in Table 2 and Appendix S1: Table S1. Seston concentration (in milligrams per liter) and the MTFA (in micrograms per milligram) had mean values (±SE) of 4.1 ± 0.4 and 5.7 ± 0.2 at PS, and 4.9 ± 0.5 and 4.8 ± 0.1 at AS. Total bacteria consisted of high nucleic acid bacteria (HNA, 73% and 56%) and low nucleic acid bacteria (LNA, 27% and 44%) at PS–AS, respectively. Picocyanobacteria (52 ± 0.3 and 34 ± 0.5 ×10^3^ cell mL^−1^) and picoeukaryotes (19 ± 2.5 and 17 ± 4.4 ×10^3^ cell mL^−1^) were the first and second predominant food sources in seston at both sites; however, concentrations of other food sources differed between them (Table 2). Fatty acid composition (FA%) was dominated by saturated fatty acids (SFA, 38%–56%), monosaturated (MUFA, 18%–31%), and polyunsaturated fatty acids (PUFA, 18%–30%) (detailed information on FA% of seston over the months in Appendix S1: Table S1). Trophic markers such as bacteria and degraded organic matter comprised more than 40% of seston available at PS and AS sites across stations (Table 2) and throughout months (Appendix S1: Table S3). Diatoms and dinoflagellates represented nearly 18%–26% in the PS and 15%–18% in the AS, suggesting that microalgae rich in eicosapentaenoic acid (EPA, 20:5ω3) and docosahexaenoic acid (DHA, 22:6ω3) were available for recruits at both sites. Green and brown macroalgae together accounted for 15%–19% in the PS and 17%–19% in the AS, indicating an important contribution to the organic matter source at both sites (Table 2).

**TABLE 2:** Mean (±SEM) of food quantity (total particulate matter, TPM, *n* = 8) and quality (mass of total fatty acids, MFTA—*n* = 6 or 8, food sources—*n* = 3 or 4, trophic markers) at stations D1, D2, and D3 in the pristine (PS) and anthropized (AS) sites during our sampling period from July to October 2021.

Food quantity and quality	PS	AS
TPM (mg L^−1^)	4.1 ± 0.4	4.9 ± 0.5
MTFA (μg mg^−1^)	5.7 ± 0.2	4.8 ± 0.1
Total bacteria (×10^6^ cell mL^−1^)	2.5 ± 0.2	1.0 ± 0.1
Pico cyanobacteria (×10^3^ cell mL^−1^)	52 ± 0.3	34 ± 0.5
Nano cyanobacteria (×10^3^ cell mL^−1^)	0.1 ± 0.0	0.04 ± 0.0
Pico eukaryotes (×10^3^ cell mL^−1^)	19.4 ± 2.5	17.1 ± 4.4
Nano eukaryotes (×10^3^ cell mL^−1^)	3.5 ± 0.2	3.7 ± 0.3
Bacteria	9.9 ± 0.1	9.8 ± 0.1
Degraded organic matter	37.2 ± 1.2	46.1 ± 0.9
Diatoms	16.5 ± 0.4	10.4 ± 0.4
Dinoflagellates	5.4 ± 0.3	6.4 ± 0.5
Green macroalgae	3.3 ± 0.1	4.4 ± 0.2
Brown macroalgae	10.2 ± 0.4	9.1 ± 0.6

### Acoustic characterization of pristine and anthropized sites

**FIGURE 3:**
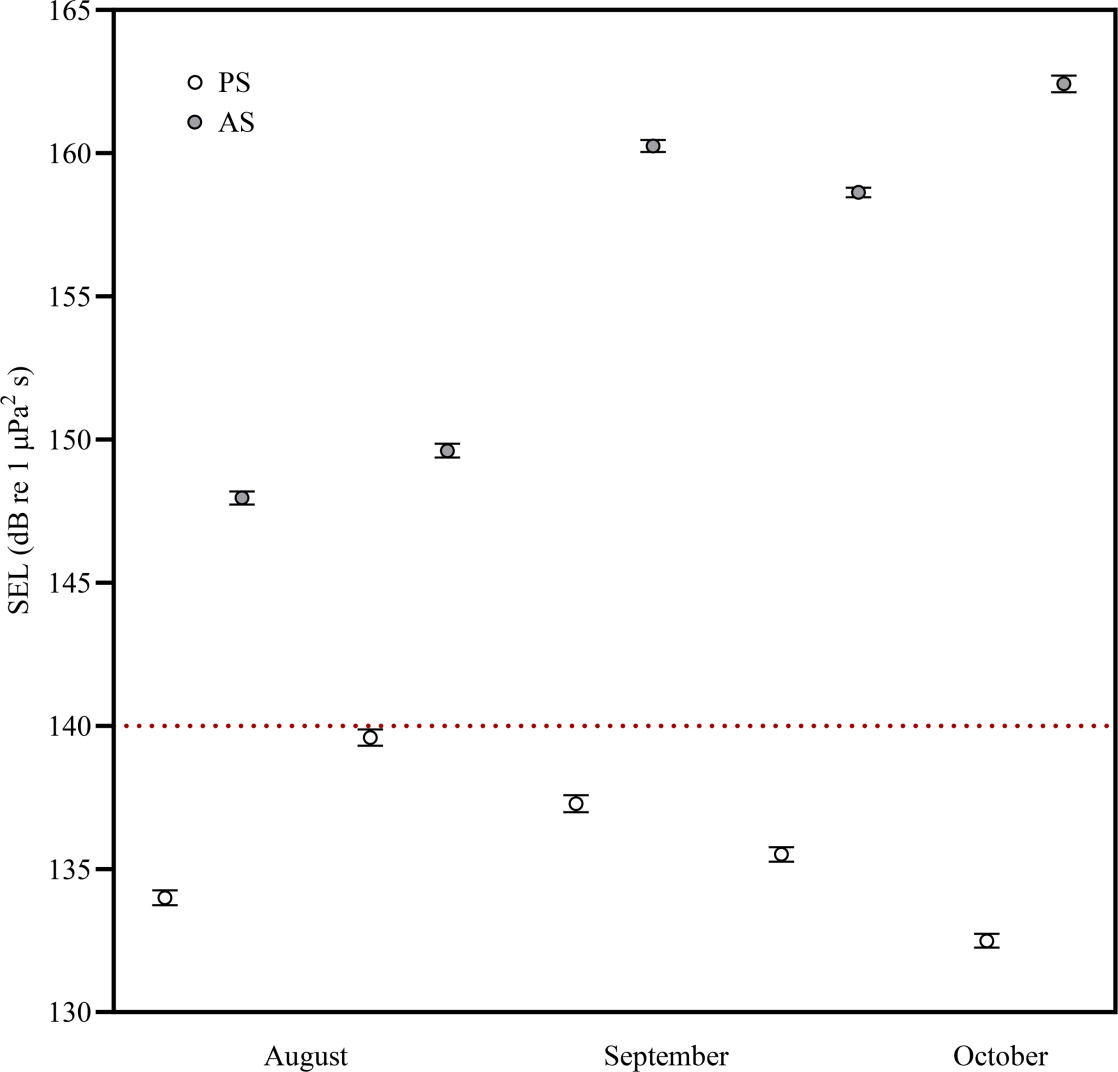
Comparison of mean (±SE) of sound exposure level (SEL_1min_, dB re 1 μPa^2^ s) experienced by invertebrates at pristine (PS—white symbol) and anthropized (AS—gray symbol) sites during August, September, and October. The red‐dashed line indicates the proposed noise threshold.

Overall, theoretical calculations (SPL_rms_, 106–137 dB re 1 μPa) and mean SEL_1min_ levels (±SE, 132 ± 0.2–138 ± 0.3 dB re 1 μPa^2^ s) at the PS site were similar throughout the months (Table 1, Appendix S1: Figure S3). In contrast, the acoustic scenario at the AS site changed completely due to pile driving operations during the same period (from 9 June to 19 October 2021), making it impossible to distinguish the vessel noise being emitted, resulting in a mixture of both. Noise levels at the AS site ranged from 143 to 154 dB re 1 μPa (SPL_pk_, Table 1), with monthly SEL_1min_ levels (mean ± SE) received by the hydrophone superior to 144 ± 0.3 dB re 1 μPa^2^ s, with maximum levels reaching 165 dB μPa^2^ s (Appendix S1: Figure S3). Maximum SEL_1min_ levels surpassed 160 dB re 1 μPa^2^ s. SEL differences between the PS and AS sites were a minimum of 9 dB re 1 μPa^2^ s in August, September, and October (Figure 3).

### Community diversity and community structure in response to anthropogenic noise

**FIGURE 4:**
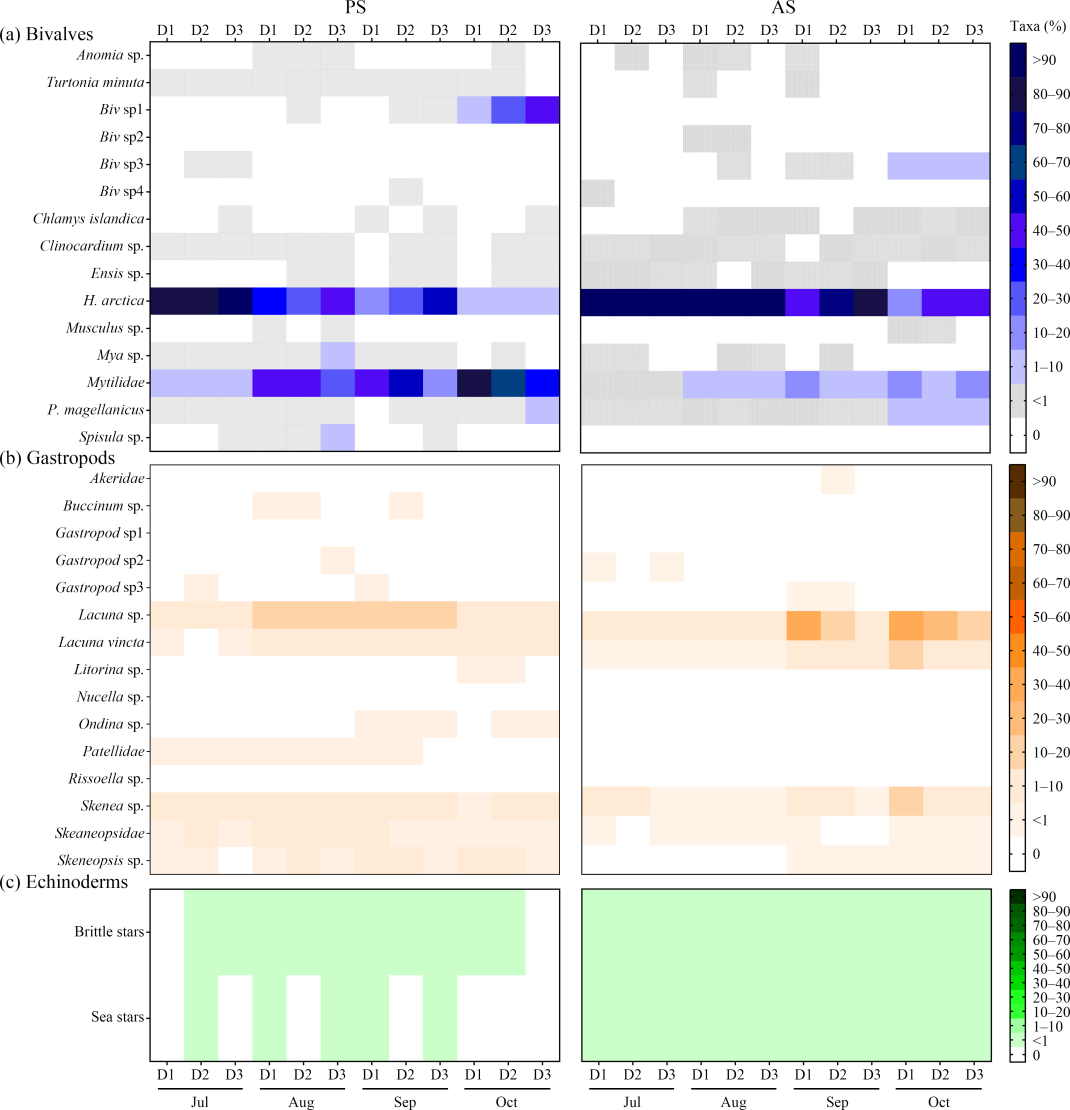
Heatmap of bivalve (blue gradient, a), gastropod (orange‐brown gradient, b), and echinoderm (green gradient, c) taxa found in artificial collectors at D1 (25–30 m), D2 (144–175 m), and D3 (848–890 m) distances from anthropogenic sounds in both experimental sites pristine (PS) and anthropized (AS) from July to October 2021.

We identified a total of 373,099 and 486,134 individuals over a 4‐month monitoring period in both sites. Thirty‐two taxa were identified across all combined stations (D1–D3) from the noise source in both sites (Figure 4a–c), with bivalves and gastropods comprising the majority of the catch (Figure 4a–c).

**FIGURE 5:**
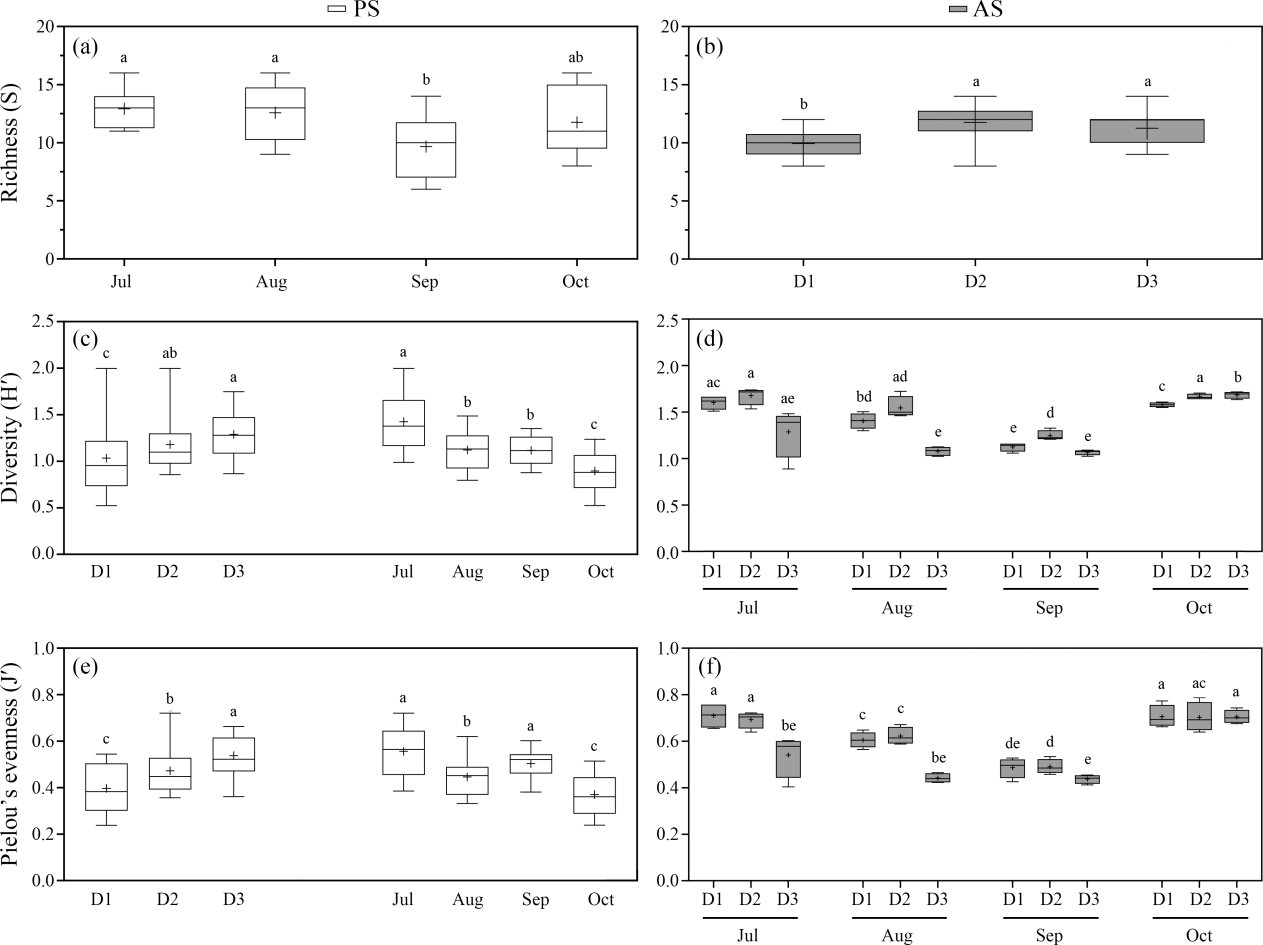
Box plot of species richness (*S*), Shannon–Wiener diversity (*H*′), and Pielou's evenness (*J*′) of assemblages obtained from collectors placed at different stations: D1 (25–30 m), D2 (144–175 m), and D3 (848–890 m) near anthropogenic noise sources in the pristine (PS, a, c, e) or anthropized (AS, b, d, f) site across 4 months—July, August, September, and October 2021. Boxes represent 25th and 75th quartiles. Whiskers show minimum and maximum values. Solid line is median, symbol plus “+” is the mean. Dissimilar letters indicate significant difference in post hoc analysis.

Diversity indices (*S*, *H*′ and *J*′) displayed distinct patterns at each site (Table 3). In the PS site, species richness significantly decreased over time, with no distance or interaction effects (Table 3, Figure 5a,c). Fewer taxa were recorded in September compared to July (*t* = 3.28, *p* = 0.004, perms = 6641) and August (*t* = 2.60, *p* = 0.02, perms = 9755). Diversity and evenness both increased with distance from the source but declined over time, with no significant interaction of factors (Table 3, Figure 5c,e; detailed in Appendix S1: Table S4). The lowest values for both diversity and evenness were observed at the highest noise level (137 dB re 1 μPa at 25 m, *H*′ = 0.95, *J*′ = 0.39) compared to moderate and control levels (120 vs. 106 dB re 1 μPa, *H*′ = 1.18, *J*′ = 0.47; *H*′ = 1.28, *J*′ = 0.54). The highest diversity was recorded in July (*H*′ = 1.42), while evenness peaked in both July and September (*J*′ = 0.55 and 0.50).

**TABLE 3:** Results of two‐way permutational multivariate analyses (PERMANOVA) testing the effect of station distances (Di, 3 levels), month (Mo, 4 levels) and their interaction (Di × Mo) on species richness (*S*), Shannon–Weiner diversity (*H*′), and Pielou's evenness (*J*′) on the assemblages of bivalves, gastropods and echinoderms retrieved from collectors moored at different distances on transects at pristine site (PS) and at anthropized site (AS).

Sites	Species richness (*S*)				Shannon–Wiener diversity (*H*′)				Pielou's evenness (*J*′)			
	df	Pseudo‐*F*	*p* (Monte Carlo)	Perm	df	Pseudo‐*F*	*p* (Monte Carlo)	Perm	df	Pseudo‐*F*	*p* (Monte Carlo)	Perm
PS												
Di	2	1.08	0.35	9952	2	16.01	**0.0002**	9961	2	21.80	**0.0001**	9956
Mo	3	4.19	**0.01**	9953	3	19.19	**0.0001**	9961	3	21.29	**0.0001**	9947
Di × Mo	6	0.87	0.53	9944	6	2.08	0.08	9955	6	2.27	0.06	9936
AS												
Di	2	7.50	**0.002**	9963	2	25.56	**0.0001**	9965	2	22.16	**0.0001**	9953
Mo	3	1.68	0.19	9935	3	55.63	**0.0001**	9954	3	57.07	**0.0001**	9963
Di × Mo	6	0.38	0.97	9941	6	6.13	**0.0008**	9957	6	4.37	**0.001**	9942

*Note*: Significant values (*p* < 0.05) are indicated in bold.

At the AS site, species richness slightly varied only by distance from the noise source, with lowest levels at highest intensity noise (D1, 153 dB re 1 μPa at 30 m) compared to other high intensities (D2, 150 dB re 1 μPa at 144 m; D3, 143 dB re 1 μPa at 848 m; *S* = 12 and 11, Figure 5b, Table 3). Diversity and evenness showed a significant interaction between distance and month, with the lowest values at D3 in July and August (*H*′ ≤ 1.28; *J*′ = 0.44–0.54). In September, both metrics declined across all distances (*H*′ < 1.24; *J*′ < 0.48, Figure 5d,f), followed by a stabilization in evenness across distances (*J*′ = 0.70), and a slight increase in diversity across intensities (*H*′ = 1.57–1.68; Figure 5d).

The community composition varied in interaction with distance and month in both PS (df = 6, pseudo‐*F*_Di×Mo_ = 5.82, *p* < 0.001, perms = 9910) and AS sites (df = 6, pseudo‐*F*_Di×Mo_ = 16.70, *p* < 0.001, perms = 9928). In the PS, community composition at high‐intensity noise (D1) differed from moderate‐low intensities (D2–D3), especially between D1 and D3 (SIMPER, up to 62% dissimilarity) in almost all periods (Appendix S1: Table S5). At AS, community also varied near high‐intensity pile driving (D1) from other higher intensities (D2–D3) (Appendix S1: Table S5), with also a pronounced distance effect between D1 and D3 and dissimilarity (SIMPER, up to 74%). SIMPER analysis showed that these differences were driven by *H. arctica* and/or Mytilidae, which were dominant taxa across months in both sites and contributed to 60%–80% of dissimilarities. Assemblages varied significantly across months at all distances in the PS and AS, though some similar compositions occurred in the AS, especially at D1 between July and August and D2–D3 between August and September (Appendix S1: Table S5).

### Species response to noise in both sites

#### H. arctica

**FIGURE 6:**
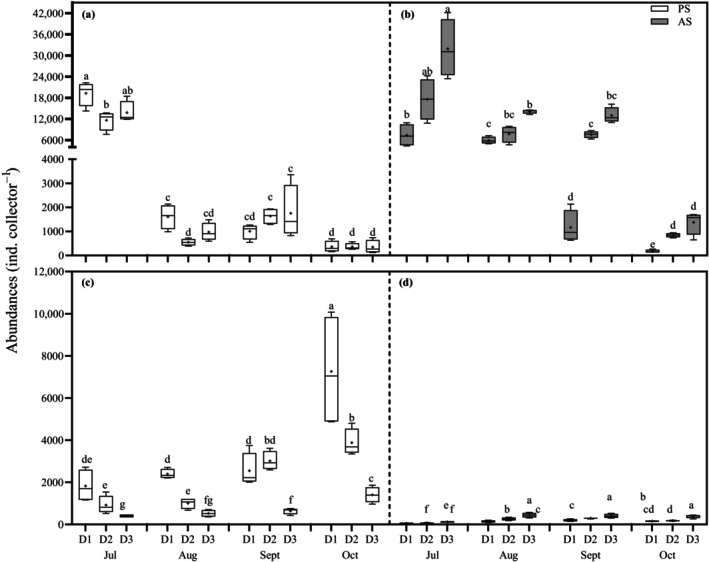
Box plots of abundances of the two most dominant bivalves *Hiatella arctica* (a, b) and Mytilidae (c, d) at different distances (D1, 25–30 m; D2, 144–175 m; and D3, 848–890 m) from anthropogenic sounds during our experimental period from July to October in the pristine (PS, a, c) and anthropized sites (AS, b, d). Boxes represent 25th and 75th quartiles. Whiskers show minimum and maximum values. Solid line is median, symbol plus “+” is mean. Dissimilar letters indicate significant difference in post hoc analysis.

In the PS site, *H. arctica* abundance varied significantly, displaying a notable interaction between distance from the noise source and month (PERMANOVA, df = 6, pseudo‐*F*_Di×Mo_ = 2.90, *p* = 0.004, Figure 6a). While the distance effect on *Hiatella* recruitment was not as pronounced, there was a clear temporal trend: recruitment peaked in July, reaching up to 19,260 ± 1748 individuals per collector in D1 (SPL_rms_, 137 dB re 1 μPa, Figure 6a). In subsequent months, abundances dropped sharply (<2000 ind. collector^−1^) and showed no significant variation across distances (Appendix S1: Table S6). In the AS site, both distance from the noise and month interactively inhibited *H. arctica* recruitment (df = 6, pseudo‐*F*_Di×Mo_ = 21.68, *p* < 0.001, Figure 6b). Abundances increased with distance from the noise, and they were 2–12 times higher at D3 (SPL_pk_, 143 dB re 1 μPa, 1370–31,899 individuals) compared to D1 across all months (153 dB re 1 μPa, 165–7347 individuals; Figure 6b). As in the PS site, recruitment peaked in July for all distances from the source, followed by a sharp reduction from August to October (Figure 6b, refer to Appendix S1: Table S6).

#### Mytilidae

In contrast to *H. arctica*, patterns of early recruitment were much clearer according to distance but also opposite between sites. In both sites, the abundance of recruits of Mytilidae was influenced by the distance from the source and month (PS, PERMANOVA, df = 6, pseudo‐*F*_Di×Mo_ = 9.76, *p* < 0.001; AS, df = 6, pseudo‐*F*_Di×Mo_ = 7.17, *p* < 0.001). In the PS, recruits were notably less abundant in the collectors moored at D3 (SPL_rms_, 106 dB re 1 μPa, 398–1396 individuals) in comparison with D1 and D2 (137 vs. 120 dB re 1 μPa, 1814–7253 individuals vs. 913–3873 individuals, please refer to Appendix S1: Table S6). The most pronounced decrease occurred between D1 and D3 in October, coinciding with the peak abundance of Mytilidae (7253 vs. 1396, Figure 6c). Throughout the early recruitment season, Mytilidae abundances gradually increased over time so that they were 4.5 to 5.0‐fold higher (>400%) at D1 when compared to D3 (Figure 6c). Over the monitoring period, recruit abundances showed a 4‐ to 5‐fold increase among intensities in the AS site (SPL_pk_, 153 dB re 1 μPa, 46–186 individuals; 150 dB re 1 μPa: 61–288 individuals; 143 dB re 1 μPa: 112–433 individuals), suggesting a clear avoidance of noisy conditions. In general, abundances were much lower in July (46–186 individuals, Figure 6d, detailed in Appendix S1: Table S6) than in the other months.

#### P. magellanicus

**FIGURE 7:**
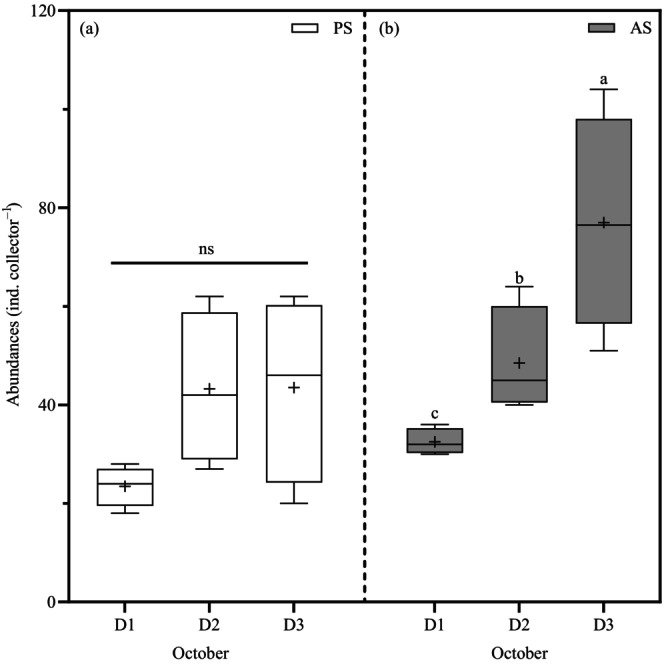
Box plots of *Placopecten magellanicus* abundances in pristine (PS, a) and anthropized (AS, b) sites per distances from the sound (D1, 25–30 m; D2, 144–175 m; and D3, 848–890 m) over October. Boxes represent lower (25%) and upper (75%) quartiles. Whiskers show minimum and maximum values. Solid line is median, and the mean is represented by the symbol plus “+.” Dissimilar letters indicate significant difference in post hoc analysis, and ns refers to non‐significant results.

*P. magellanicus* recruits were only collected in October in both sites, and their abundance was similar between distance in the PS (SPL_rms_, 23–43 individuals, PERMANOVA, df = 2, pseudo‐*F*_Di_ = 2.79, *p* = 0.11, Figure 7a) whereas they increased by 2‐fold with distance (SPL_pk_, df = 2, pseudo‐*F*_Di_ = 13.72, *p* = 0.001) from D1 to D3 in the AS site (153–143 dB re 1 μPa, 32, 48, 77 individuals, *t*_D1–D3_ = 5.14, *p* = 0.001; *t*_D2–D3_ = 2.391, *p* = 0.048; Figure 7b).

#### *Lacuna* sp.

**FIGURE 8:**
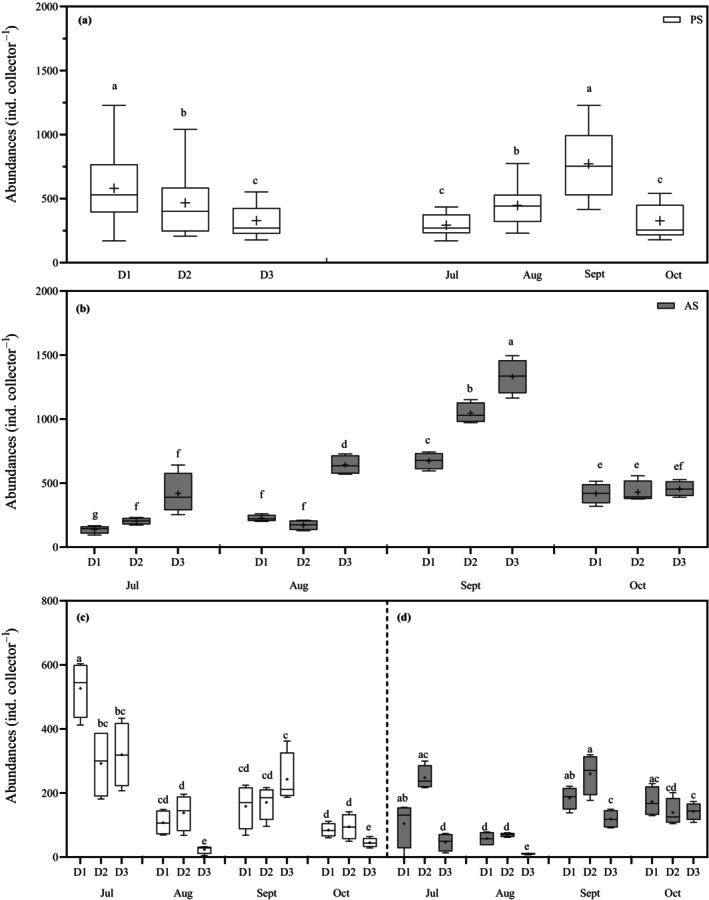
Box plots of (a, b) *Lacuna* sp. and (c, d) *Skenea* sp. abundances per station from the sound (D1, 25–30 m; D2, 144–175 m; and D3, 848–890 m) during our experimental period from July to October 2021 in the pristine site (PS) and anthropic site (AS). Boxes show 25th and 75th quartiles, while whiskers show minimum and maximum values. Solid line is median; symbol “+” denotes the mean. Dissimilar letters indicate significant difference in post hoc analysis.

In PS, *Lacuna* sp. recruitment was influenced by both distance from source (PERMANOVA, df = 2, pseudo‐*F*_Di_ = 11.47, *p* = 0.001) and month (df = 3, pseudo‐*F*_Mo_ = 24.37, *p* = 0.001) without interaction of factors (Figure 8a). Recruit abundance decreased from 580 to 329 ind. collector^−1^ across D1 to D3 (SPL_rms_, 137 dB–106 dB re 1 μPa, *t*_D1–D2_ = 2.02, *p* = 0.04; *t*_D1–D3_ = 5.12, *p* < 0.001; *t*_D2–D3_ = 2.68, *p* = 0.01, Figure 8a) peaking in September (771 individuals, *t*_Jul–Sept_ = 7.76, *p* < 0.001; *t*_Aug–Sept_ = 5.121, *p* < 0.001; *t*_Sept–Oct_ = 7.583, *p* < 0.001). In the AS site, the pattern was inverse, and there was a significant interaction between both factors for *Lacuna* sp. abundances (df = 6, pseudo‐*F*_Di×Mo_ = 15.325, *p* < 0.001). In this site, recruit abundance was ~3‐fold higher at D3 than at D1 across months (SPL_pk_ 140 vs. 153 dB re 1 μPa; 136 vs. 417, 225 vs. 641, 673 vs. 1331 individuals), except in October when abundances were similar among distances. The highest peak of early recruitment was observed in September (Figure 8b, Appendix S1: Table S6).

#### *Skenea* sp.

*Skenea* sp. abundances varied significantly with distance and month at both sites (details in Appendix S1: Table S6) (PS: PERMANOVA, df = 6, pseudo‐*F*_Di×Mo_ = 3.97, *p* = 0.001, perms = 9940; AS: df = 6, pseudo‐*F*_Di×Mo_ = 16.57, *p* < 0.001, perms = 9932, Figure 8c,d). In the PS, abundances decreased with distance from the noise source and over time (526 vs. 319, 107 vs. 23 individuals). Overall, higher abundances were firstly observed in D1, and later in D1–D2 (SPL_rms_, 137–120 dB re 1 μPa, Figure 8c). In the AS, D1 and D2 consistently showed 2‐ to 3‐fold higher abundances compared to D3 (SPL_pk_, 153–150 vs. 143 dB re 1 μPa, 104 and 247 vs. 46 individuals, 184 and 259 vs. 118 individuals, Figure 8d), suggesting a possible stimulation effect on D1–D2 versus D3 over 3 months. Temporal patterns showed that *Skenea* sp. recruitment peaked at different times across noise intensities: D1–D2 had similar high abundances in July and September (184 vs. 172, 247 vs. 259, D1–D2), while D3 peaked later, between September and October (118–142 individuals).

## DISCUSSION

To our knowledge, the present study provides the first field evidence of ecological shifts at the community and species level during early recruitment in response to anthropogenic noise.

### Pristine and anthropized sites differ in environmental conditions and soundscapes

The SPM archipelago experiences extreme thermal oscillations, with temperatures shifting by up to 11°C in 90 min during September, driven by nonlinear internal waves amplified by diurnal tides (Lazure et al., 2018). These fluctuations are more pronounced at 30–60 m depths than in shallower waters (<20 m). Temperature conditions were homogeneous across stations within each site, and seasonal shifts were evident, aligning with previous studies (Bridier et al., 2021; Lazure et al., 2018; Poitevin et al., 2020). Given that temperature is a key factor regulating the physiology and behavior of bivalves and gastropods (Bayne, 1965; Pechenik, 1990; Tremblay et al., 1998), short and intense thermal events caused by internal waves may impose significant stress on recruits (Garwood et al., 2020; Woodson, 2018), likely in the AS. These site‐specific dynamics highlight the complex relationship between temperature and bivalve development in SPM (Poitevin et al., 2020).

Hydrodynamic conditions, including turbulence and current flow, influence larval behavior and distribution, and ultimately settlement, with bivalves and gastropods showing preferences for water motion and turbulence depending on their habitat (Fuchs et al., 2018; Pernet et al., 2003; Tremblay et al., 2020). In addition, to expose larvae to temporary thermal stress, internal waves can affect larval transport, potentially facilitating larvae's final delivery to settlement sites (see references and discussion in Garwood et al., 2020; Woodson, 2018). In this study, weak bottom and residual currents likely promoted recruit retention during the early months along both transects. After mid‐September, bottom current intensities peaked at the AS site, coinciding with a stronger thermal gradient (7–10°C), possibly linked to internal wave activity in August–September. These changes may have disrupted larval delivery, with fewer larvae reaching stations across the transect at the AS site. Furthermore, inshore current patterns did not favor recruit accumulation near collectors close to the noise source, as depicted by ADCP figures (D1, refer to Appendix S1: Figure S1b–d).

The trophic environment plays a key role in supporting larval development and recruitment by supplying nutritional resources and acting as a cue, synchronizing settlement–recruitment events, as observed in *Mytilus edulis*, Mytilidae, and *Crassostrea gigas* in temperate habitats (Androuin et al., 2022; Lagarde et al., 2018; Toupoint, Gilmore‐Solomon, et al., 2012). Consistent with Bridier et al. (2021) and Toupoint, Gilmore‐Solomon, et al. (2012), the pelagic trophic environment in SPM, including PS and AS, is oligotrophic. This was supported by the high proportions of bacterial and degraded organic matter markers (>40%, Table 2) and elevated bacterial concentrations in the potential food source (1.0–2.5 × 10^6^ cells mL^−1^, Table 2). Flow cytometry data indicated a dominance of picoplankton (picocyanobacteria and picoeukaryotes, Table 2, and Appendix S1: Table S3) over nanoplankton at both sites, likely contributing to the diet of local filter‐feeders. Food quantity (TPM) and quality available (MFTA, trophic markers, food sources) were consistent across distances at each site. However, the PS site showed greater food availability and quality than the AS site, including higher proportions of diatom markers and evidence of diatom blooms in September and October, confirming secondary autumnal blooms in SPM (Bridier et al., 2021). Importantly, similar trophic conditions across distances ruled out trophic variation as a confounding factor when assessing the effects of noise in our trials.

Soundscapes differed markedly between the PS site (vessel noise, SPL_rms_ = 106–137 dB re 1 μPa) and AS site (mix of pile driving and vessel noise, SPL_pk_ > 140 dB re 1 μPa), where a combination of pile driving and vessel noise produced higher sound levels. Correspondingly, organisms were exposed to different sound exposure levels (PS: 132–138 dB re 1 μPa^2^ s vs. AS: >140 dB re 1 μPa^2^ s). Recent research shows that impulsive and continuous noises differ acoustically and can reduce habitat sensitivity, causing varied behavioral and physiological effects depending on sound pressure, frequency, and distance from source (for review, see Bonnel et al., 2022; Solé et al., 2023). Given that both physical and trophic conditions were overall consistent across sampling distances within each site, the observed patterns (species distribution, settlement, and early recruitment) are more likely attributed to the noise gradient rather than environmental variability.

### Impact of noise on biodiversity features of recruit assemblages

In the past years, researchers have increasingly underlined the need for field‐based assessments of anthropogenic impacts on marine ecosystems. Yet, significant gaps remain in understanding how anthropogenic noise affects biodiversity and population dynamics (Kunc et al., 2016; Solé et al., 2023; Sordello et al., 2020). This study provides the first field evidence of invertebrate community shifts due to anthropogenic noise exposure. Recruit diversity and evenness shifts aligned with the nature and intensity of the noise source: introducing vessel noise at the PS site (137 dB re 1 μPa, SEL < 132–138 dB 1 μPa^2^ s) led to lower diversity and evenness (*H*′ and *J*′) near the source favoring stress‐tolerant species, while higher intensity mixed noises at the AS site (SPL_pk_ > 143 dB re 1 μPa, SEL > 140 dB re 1 μPa^2^ s) were associated with differences in *H*′ and *J*′ indices, greater species turnover, and higher diversity at moderate intensity (D2) due to rare species in August and October. Such diversity increase at D2 could be in line with the *intermediate disturbance hypothesis (IDH)* proposed by Connell (1978) where diversity is highest at intermediate levels of disturbance due to the trade‐off in the presence of common and rare species. We suggest that a threshold below <140 dB re 1 μPa^2^ s is required to avoid changes in community structure.

While terrestrial studies report declines in bird species richness under anthropogenic traffic noise (Perillo et al., 2017; Proppe et al., 2013), and unchanged biodiversity of amphibians under turbine audio recordings at 500 m (Trowbridge & Litzgus, 2022), such comparisons are limited given the propagation of sound in water (Bonnel et al., 2022; Farina, 2014; NRC, 2003). Despite the growing interest and urgent need to develop threshold criteria based on sound pressure level and sound exposure thresholds (SPL,dB re 1 μPa and SEL, dB re 1 μPa^2^ s) to prevent several biological effects in marine fauna, frameworks connecting noise disturbances and impulsive and continuous noises have been mostly developed for marine mammals, and to an extent for fish (see Bonnel et al., 2022 for a detailed review; NFMS, 2018; NRC, 2003; Popper et al., 2014). In general, behavior modifications are seen in different groups of marine mammals when SPL and SEL of impulsive sounds are above 224 dB re 1 μPa and 173 dB re 1 μPa^2^ s (references in Bonnel et al., 2022, p. 84). Auditory effects such as temporary threshold shift (TTS) in different hearing groups may occur if continuous sounds are above 153 dB re 1 μPa^2^ s (SEL_cum_), with higher SPL and SEL thresholds for impulsive noises like pile driving (196 dB re 1 μPa and 140 dB re 1 μPa^2^ s) (references cited within Bonnel et al., 2022, p. 86; NMFS, 2018). Fish injury thresholds, depending on hearing group, are set at 183–187 dB re 1 μPa^2^ s (SEL_cum_) and 206  dB re 1 μPa (SPL_pk_), while TTS is above 186 dB re 1 μPa^2^ s (SEL) for pile driving. For fish behavioral effects under ship or pile driving exposure, the threshold is set at 150 dB re 1 μPa, uncertain if it is SPL_pk_ or SPL_rms_ (Popper et al., 2014, p. 35).

Community diversity and stability depend on recruitment processes such as predation and competition (Austen et al., 2002; Butman, 1987; Fraschetti et al., 2003), and secondary migrations—key mechanisms in the movement of *M. edulis*, *H. arctica*, and *Lacuna vincta* (Forêt et al., 2018; Le Corre et al., 2013; Martel & Chia, 1991; Veillard et al., 2023). In this 4‐month study, we accounted for these post‐settlement processes (Hunt & Scheibling, 1997). Predation, a major source of settler mortality (Beal et al., 2020; Gosselin & Qian, 1997; O'Connor et al., 2008), was likely minimal here due to the scarcity (<10%) or absence of sea stars and crabs. Interspecific competition, often affecting growth and survival in aquaculture collectors (Cyr et al., 2007; Garcia et al., 2003; Khalaman, 2005), likely had little effect due to the short deployment (1‐month). Although noise may disrupt both predation and competition (Chan et al., 2010; Roberts et al., 2015), its role remains unclear in situ. However, we noticed a high proportion of larger recruits (10%–80%, >500 μm) of Mytilidae and *Lacuna* sp. were concentrated near the noise source (137 dB re 1 μPa) in the PS site, suggesting that vessel noise may trigger secondary migrations. Conversely, in the AS site, larger recruits of *H. arctica*, Mytilidae, and *P*. *magellanicus* were more abundant farther from the pile driving source (143 dB re 1 μPa, 40%–90%, species‐dependent), supporting that mixed noises may differently influence post‐settlement movement. Further research is needed to understand how anthropogenic noise shapes post‐settlement dispersal.

Given the species‐specific responses noticed in this study, we present site‐specific findings to showcase contrasting patterns and propose potential sound pressure and noise exposure thresholds that may elicit invertebrate behavioral responses.

### Should I stay or should I go? Toward identifying a noise threshold for recruitment

Early studies with oysters suggest that the exposure to acoustic cues may alter developmental trajectories or trigger physiological responses that interact with other settlement cues. A recent study has demonstrated that this orientation in oyster larvae may go beyond vertical orientation, as larvae exhibited horizontal movements along a reef sound gradient, in field and lab studies (Williams et al., 2022). Research also suggests that larvae may prioritize certain cues—pulse of phytoplankton versus biofilm (Toupoint, Gilmore‐Solomon, et al., 2012; Toupoint, Mohit, et al., 2012)—or interpret them as positive or negative, as with algae versus predator (Morello & Yund, 2016). Because vessel noise and pile driving fall within invertebrate hearing ranges (Chauvaud et al., 2018; Duarte et al., 2021), they can mask critical ambient cues and interfere with larval behaviors during habitat selection and settlement (Fuchs et al., 2018; Kingsford et al., 2002; Lillis et al., 2013, 2015).

### Pristine site versus anthropized site

In this study, early recruitment patterns were strongly influenced by the anthropogenic soundscape, with site‐specific shifts corresponding to SPL and SEL thresholds. At the pristine site (PS), vessel SPLs decreased from high‐moderate intensities (137–120 dB re 1 μPa) to control (106 dB re 1 μPa), and SELs remained below 138 dB re 1 μPa^2^ s. Under these high‐moderate conditions, recruitment of Mytilidae, *H. arctica*, *Lacuna* sp., *and Skenea* sp. was 3‐ to 5‐fold enhanced, while trends of *P. magellanicus* were inconclusive due to limited sampling. The similar responses among most taxa here suggest that a threshold of 132–138 dB re 1 μPa^2^ s may elicit behavioral change, increasing early recruitment near the vessel noise source. In contrast, the anthropized site (AS) experienced cumulative noise from pile driving and vessel noise, with SPLs exceeding 140 dB re 1 μPa and SELs above 140 dB re 1 μPa^2^ s. In these circumstances, recruitment of most taxa declined 2‐ to 3‐fold, except *Skenea* sp., which increased by a similar magnitude.

While studies on bivalve or gastropod settlement under noise exposure remain scarce in situ, insights can be drawn from laboratory‐based trials about the behavior under certain vessel noise or pile driving exposure (SPL_rms_, SPL_pk_). The stimulation pattern seen in bivalve and gastropods aligns with the following studies. Veillard et al. (2025) observed a 2‐fold increase in mussel settlement and metamorphosis under higher vessel noise compared to control levels (SPL_rms_, 151 vs. 116 dB re 1 μPa) using the *Larvosonic* system, which simulates a sound gradient from the sound source (see Olivier et al., 2023 for details). This suggests a similar behavioral shift as in the PS. In contrast, Jolivet et al. (2016) revealed a 27% increase after 67 h of noise exposure (127 dB re 1 μPa, 100–1000 Hz), while Cervello et al. (2023) found no differences between control and boat noise treatments (128 and 139 dB re 1 μPa). However, the differences in these two studies may stem from the fact that the first experiments were conducted in tanks where several acoustical biases can occur such as resonance and reverberations, whereas the absence of water motion as in the natural environment was one of the main issues in Cervello et al. (2023). The slight stimulation of *H. arctica* abundances toward vessel noise at the PS is a new result since there is no research using this species as a biological model for noise impact. Given the habitat overlap of mytilids and *Hiatella* (Garcia et al., 2003), we suggest that similar behavioral effects may occur to noise threshold at the PS.

For gastropods, few studies exist—Stocks et al. (2012) reported increases in swimming activity despite not describing sound levels, while Solé et al. (2021) documented statocyst damage at a wave sweep of 157 dB 1 μPa (50–400 Hz). As *Lacuna* sp. recruitment increased near high‐moderate (120–137 dB re 1 μPa, D1–D2) at PS, we hypothesize that their presence near the source may reflect a behavioral threshold near 132–138 dB re 1 μPa^2^ s in the PS.

For pile driving sources in the anthropized site (AS), Cervello et al. (2023) noticed a downward trend in mussel settlement under pile driving and control treatments in *Larvosonic* trials (166.4 vs. 128 dB re 1 μPa), though their results were not statistically significant. Scallop recruitment also declined near pile driving sources (>150 dB re 1 μPa), contrasting with controlled studies using a pectinid, *Pecten maximus*, which showed no survival or metamorphosis declines at even higher pile driving sounds (*Larvosonic*, SPL_pp_ 148–188 re 1 μPa, Gigot, Olivier, et al., 2023; Olivier et al., 2023). Nonetheless, stress responses have been recorded in *P. magellanicus* juveniles in situ at lower SEL (SEL_ss_ < 94.39 dB re 1 μm s^−2^ at 8 m), suggesting that our higher exposures (SEL > 140 dB re 1 μPa^2^ s) may have triggered similar responses that potentially interfered with settlement or feeding (Robson et al., 2012). Likewise, *Lacuna* sp. abundances declined under higher intensities (150–153 dB re 1 μPa); we propose that abundance decreases at D1–D2 may result from auditory damage at higher exposure (above 140 dB re 1 μPa^2^ s), consistent with auditory damage observed by Solé et al. (2021).

Though the mechanism of hearing in invertebrates remains poorly understood (Chauvaud et al., 2018), evidence suggests that they detect particle motion via statocysts, allowing perception and orientation toward sound sources (Hawkins & Popper, 2017). This sensory system becomes more complex after metamorphosis and involves different structures at specific life stages (references in Solé et al., 2023; Zhadan, 2005). In addition to statocysts in the ciliated foot, superficial mechanoreceptors on the epidermis work as accelerometers (Budelmann, 1992; Cragg & Nott, 1977; Roberts et al., 2015).

Several potential mechanisms may underlie the observed responses to vessel noise and mixed noises. Beyond sound intensity, frequency is critical, as many invertebrates produce and perceive a range of sounds that overlap natural and anthropogenic sources (Chauvaud et al., 2018; Roberts et al., 2015; Zhadan, 2005). In this study, the low‐frequency vessel sound may have mimicked some natural cues from rocky shores/waves, attracting larvae, as initially proposed by Jolivet et al. (2016). Additionally, in the absence of physical, chemical, or natural acoustic cues, anthropogenic sounds (e.g., vessel) may fill in as substitute signals with similar acoustic signatures, modifying larval behavior and accelerating this transition. However, when vessel and pile driving noises were combined, the resulting higher intensity and threshold may have indicated suboptimal conditions for most taxa, as strong substrate‐borne vibrations and particle motion near the source could interfere with valve closure or foot extension, as suggested by Jézéquel et al. (2022) and Roberts et al. (2015).

Considering our findings, we suggest a sound exposure threshold not exceeding 140 dB re 1 μPa^2^ s as a potential upper limit for maintaining normal recruitment patterns of key invertebrate species. Below this level (132–138 dB re 1 μPa^2^ s), early recruitment of several species was enhanced or unaffected, which needs to be interpreted with caution here as this could impair selectivity and increase metabolic stress in post‐metamorphic stages (Veillard et al., 2025). While these thresholds are broadly consistent with preliminary criteria for marine mammals and fishes (Bonnel et al., 2022; NMFS, 2018; Popper et al., 2014), more studies should be carried out to obtain complete dose–response curves and validate these proposed levels.

## FUTURE PERSPECTIVES

This study demonstrates that anthropogenic noise alters marine community diversity and evenness, affecting recruitment of stress‐tolerant species in situ depending on noise nature and thresholds. This is the first field study to examine the effect of noise on marine community traits and propose a preliminary threshold for invertebrates (<140 dB re 1 μPa^2^ s). While several gaps remain unaddressed in noise impact studies (Duarte et al., 2021; Hawkins & Popper, 2017; Solé et al., 2023), we highlight three areas requiring further attention:

1. Environmental stressors—such as anthropogenic soundscapes, temperature shifts, and food availability—may cause latent effects in young juveniles that persist, amplify, or compensate over time (Lagarde et al., 2018; Martel et al., 2014; Podolsky & Moran, 2006). For example, delayed metamorphosis, triggered by low temperature or insufficient trophic conditions, have been referred as amplification effects, which might result in a decrease in larval energetic reserves available to metamorphose, reduced ability to identify specific cues to settle and in the end, reduced fitness, survival, and growth in juveniles and adults (Hunt & Scheibling, 1997; Pechenik, 2006; Podolsky & Moran, 2006). Noise exposure has been pointed out as a new trigger of amplifying and compensating latent effects. Gigot et al. (2024) found that scallops (*P. maximus*) exposed to moderate pile driving noise produced fewer eggs than control condition (SPL_pp_ 148 vs. 100 dB re 1 μPa), but their larvae exhibited enhanced growth—a cross‐generational response that likely amplifies the survival of offspring. Although we did not measure size at metamorphosis, previous studies on oysters suggest that larvae settling earlier (at smaller sizes) achieved higher recruitment success (Lagarde et al., 2018). These findings highlight the need to better understand how interacting stressors may produce latent effects that compromise recruitment in the Anthropocene era.
2. In situ experiments offer greater ecological realism than laboratory studies, despite reduced experimental control (Spicer, 2014). Despite some design constraints, this study successfully accounted for major confounding factors (temperature, trophic conditions, currents) and proposes a realistic baseline threshold (>140 dB re 1 μPa^2^ s) for inhibited recruitment across taxa. This threshold may offer guidance for conservation policy. Future research should validate these levels by examining long‐term physiological response, trophic quality available in situ, temperature and ecological interactions under single and multiple noise exposures.
3. The post‐settlement phase of bivalve and gastropods—often downplayed due to high mortality—plays a critical role in population dynamics. These relocations are prompted not only by environmental conditions but also by the active behavior of post‐larvae (Forêt et al., 2018; Le Corre et al., 2013; Leal et al., 2022; Martel & Diefenbach, 1993). Noise may disrupt these secondary migrations, influencing substrate choice and habitat selection. A shift to a less suitable habitat could undermine population replenishment, particularly in disturbed intertidal zones. More research is needed to understand how noise affects this phase of the benthic life cycle.

## AUTHOR CONTRIBUTIONS

*Study conception*: Réjean Tremblay, Frédéric Olivier, and Nathália Byrro Gauthier. *Methodology*: Réjean Tremblay, Frédéric Olivier, Nathália Byrro Gauthier, Gesche Winkler, Tarik Meziane, Pascal Lazure, Delphine Mathias, Laurent Chauvaud, Antoine Frémont, and Sylvain Chauvaud. *Data collection*: Nathália Byrro Gauthier, Thomas Uboldi, and Delphine Mathias. *Data analysis*: Nathália Byrro Gauthier, Delphine Mathias, and Pascal Lazure. *Drafting the article*: Nathália Byrro Gauthier. *Review and editing*: Nathália Byrro Gauthier, Thomas Uboldi, Réjean Tremblay, Frédéric Olivier, Gesche Winkler, Tarik Meziane, Pascal Lazure, Delphine Mathias, Laurent Chauvaud, Antoine Frémont, and Sylvain Chauvaud. *Supervision*: Réjean Tremblay, Gesche Winkler, and Tarik Meziane. *Project administration*: Réjean Tremblay and Frédéric Olivier. *Funding acquisition*: Réjean Tremblay, Frédéric Olivier, and Gesche Winkler. All authors have read and approved the final version of the manuscript. This manuscript is a contribution to the strategic cluster Ressource Aquatique Québec (RAQ).

## CONFLICT OF INTEREST STATEMENT

The authors declare no conflicts of interest.

## Supporting information


Appendix S1.

## Data Availability

Data and code (Byrro Gauthier, 2025) are available in Figshare at https://doi.org/10.6084/m9.figshare.29708534.
