# Supplementary material for: Should I stay, or should I go: Anthropogenic noises disrupt early recruitment of subarctic invertebrates
Source: Ecol Appl. 2025 Nov 10;35(7):e70119. doi: 10.1002/eap.70119 (PMC12603344; doi:10.1002/eap.70119)
Supplement: Supplementary file 1 — Appendix S1. [file EAP-35-e70119-s001.pdf]

Appendix S1

Should I stay, or should I go: Anthropogenic noises disrupt early recruitment of subarctic invertebrates

Nathália Byrro Gauthier, Thomas Uboldi, Frédéric Olivier, Réjean Tremblay, Laurent Chauvaud, Delphine Mathias, Pascal Lazure, Antoine Frémont, Tarik Meziane, Sylvain Chauvaud, Gesche Winkler

Ecological Applications

Section S1: Material and Methods

Laboratory procedures

Characterization of trophic environment: Fatty acids extraction and trophic markers Filters with seston were freeze-dried in a tabletop freeze dryer Christ Alpha 1-2 LSC basic (Osterode, Germany) prior to the extraction. After that, filters were weighed (mg) using a Mettler Toledo MS digital scale, and seston concentration (TPM dry mass mg.L^{-1}) was determined. Fatty acids from seston were extracted and analyzed by Gas-Chromatography-Mass Spectrophotometry to identify all the fatty acids (%) and to quantify their total mass (MTFA, µg.mg^{-1}). Lipids were extracted following a slightly modified method of Bligh and Dyer (1959) adapted by Meziane et al. (2007). The detailed extraction protocol used is described in Chynel et al. (2022). Briefly, fatty acid quantification was performed using a Varian CP-3800 gas chromatograph equipped with a Supelco® Omegawax® Capillary GC 320 column. Fatty acid identifications were validated using retention times and mass of total fatty acids (MTFA, µg.mg^{-1}) and its quality, such as the composition (%) of SFAs, MUFAs, PUFAs spectra measured from a commercial reference standard (Supelco® 37 Component FAME Mix). Mass spectra were measured with a Varian 220-MS coupled to a Varian 450-GC. Concentration of each fatty acid peak was determined using an internal commercial standard (23:0). Fatty acid composition was further discriminated into FA trophic markers attributed to six sources (bacteria, degraded organic matter, diatoms, dinoflagellates, green macroalgae, and brown macroalgae), similar markers as Leal et al. (2022). Unfortunately, we were unable to analyze AS site's FA samples in July due to logistical issues.

Table S1: Results of one-way permutational multivariate analyses (PERMANOVA) testing the effect of distance (Di, 3 levels) on total particulate matter (TPM, $mg.L^{-1}$, n=6 or 8), fatty acid composition (%), total mass of fatty acid (MFTA, $\mu g.mg^{-1}$, n=6 or 8), trophic markers (%) and food sources (total bacteria, pico- and nano cyanobacteria, pico- and nano eukaryotes) available for recruits collected on transects at pristine (PS) and anthropized (AS) sites. Significant values (p<0.05) are indicated in bold.

	**TPM ($mg.L^{-1}$)**				**FA composition (%)**				**MFTA ($\mu g.mg^{-1}$)**			
**PS**	*df*	*Pseudo-F*	*p (MC)*	*Perm*	*df*	*Pseudo-F*	*p (MC)*	*Perm*	*df*	*Pseudo-F*	*p (MC)*	*Perm*
Di	2	0.22	0.80	9947	2	0.65	0.64	9942	2	0.42	0.74	9953
Res	21				21				21			
Total	23				23				23			
**AS**												
Di	2	0.12	0.63	9895	2	1.43	0.22	9932	2	0.36	0.78	9930
Res	15				14				14			
Total	17				16				16			
	**Trophic markers**				**Food sources**							
**PS**	*df*	*Pseudo-F*	*p (MC)*	*Perm*	*df*	*Pseudo-F*	*p (MC)*	*Perm*				
Di	2	0.73	0.5818	9941	2	1.00	0.44	1622				
Res	21				9							
Total	23				11							
**AS**												
Di	2	1.08	0.3782	9928	2	0.73	0.60	226				
Res	15				6							
Total	17				8							

Table S2: Fatty acid composition (FA%, mean ± SE, n=6) of bottom particulate organic matter (b-POM) retrieved from artificial collectors moored at distances D1 (25-30m), D2 (144-175m) and D3 (848-890m) from an underwater speaker emitting vessel sound on a transect in the pristine (PS) and anthropized (AS) site between July, August, September, and October 2021, respectively. Values are reported as traces (tr.) when percentages are inferior 0.1%. Abbreviations: branched fatty acids (brFA), saturated fatty acids (SFA), monosaturated fatty acids (MUFA) polyunsaturated fatty acids (PUFA), eicosapentaenoic acid (EPA), docosahexaenoic acid (DHA), and essential fatty acids (EFA). BrFA are composed of 13:0iso, 14:0iso, 15:0anteiso, 15:0iso and 16:0iso.

	**PS**				**AS**		
**FA**	*July*	*August*	*September*	*October*	*August*	*September*	*October*
12:0	0.45 ± 0.06	0.4 ± 0.05	0.36 ± 0.05	0.39 ± 0.09	0.44 ± 0.09	0.37 ± 0.04	0.54 ± 0.09
13:0	0.11 ± 0.01	0.13 ± 0.01	0.16 ± 0.01	0.19 ± 0.01	0.1 ± 0.02	0.1 ± 0.01	0.12 ± 0.02
14:0	6.11 ± 0.41	6.55 ± 0.31	8.62 ± 0.28	8.31 ± 0.41	10.16 ± 0.26	10.07 ± 0.21	9.04 ± 0.44
15:0	0.74 ± 0.05	1.02 ± 0.06	0.94 ± 0.02	1.34 ± 0.02	0.89 ± 0.03	0.93 ± 0.04	1.28 ± 0.03
16:0	27.92 ± 1.56	22.12 ± 0.98	19.14 ±0.21	19.79 ± 0.2	27.87 ± 1.57	26.74 ± 0.36	26.6 ± 0.93
17:0	tr.	0.12 ± 0.02	0.93 ± 0.03	0.7 ± 0.08	tr.	0.42 ± 0.01	0.08 ± 0.01
18:0	14.47 ± 2.3	7.19 ± 0.96	4.12 ± 0.07	5.21 ± 0.1	11.93 ± 1.65	6.85 ± 0.39	8.97 ± 0.49
20:0	1.25 ± 0.36	1.45 ± 0.24	3.09 ± 0.16	0.98 ± 0.14	3.62 ± 0.32	4.19 ± 0.11	3.11 ± 0.29
22:0	0.1 ± 0.02	0.19 ± 0.01	0.21 ± 0.01	0.38 ± 0.02	0.18 ± 0.02	0.15 ± 0.01	0.2 ± 0.03
24:0	0.1 ± 0.01	0.17 ± 0.03	0.18 ± 0.03	0.26 ± 0.08	0.07 ± 0.04	tr.	tr.
26:0	tr.	0.15 ± 0.08	tr.	0.27 ± 0.09	0.41 ± 0.13	tr.	tr.
**Σ SFA**	**51.38 ± 3.38**	**39.5 ± 1.94**	**37.8 ± 0.61**	**37.81 ±0.53**	**55.72 ± 2.79**	**49.93 ± 0.57**	**50.07 ± 0.51**
14:1ω3	0.16 ± 0.01	0.23 ± 0.01	0.29 ± 0.03	0.35 ± 0.04	0.19 ± 0.01	tr.	0.21 ± 0.02
16:1ω5	0.58 ± 0.04	0.36 ± 0.01	0.48 ± 0.02	0.59 ± 0.02	0.45 ± 0.02	0.57 ± 0.01	0.45 ± 0.02
16:1ω7	6.44 ± 0.41	9.95 ± 0.34	12.64 ±0.17	16.1 ± 0.59	5.21 ± 0.13	6.95 ± 0.12	8.09 ± 0.35
16:1ω9	tr.	tr.	tr.	0.1 ± 0.02	tr.	tr.	0.39 ± 0.35
17:1ω7	0.23 ± 0.03	0.22 ± 0.02	0.43 ± 0.02	0.08 ± 0.02	0.29 ± 0.05	0.37 ± 0.01	0.32 ± 0.05
18:1ω7	5.5 ± 0.31	8.53 ± 0.51	6.32 ± 0.41	10.02 ±0.32	3 ± 0.08	3.51 ± 0.13	4.11 ± 0.24
18:1ω9	7.87 ± 0.43	11.66 ± 0.32	9.05 ± 0.37	12.28 ±1.35	8.83 ± 0.83	8.4 ± 0.2	10.16 ± 0.56
**Σ MUFA**	**20.83 ± 1.19**	**31.04 ± 0.83**	**29.27 ±0.67**	**39.52 ± 0.9**	**18 ± 0.88**	**19.93 ± 0.3**	**23.74 ± 0.84**
16:2ω4	0.16 ± 0.03	0.22 ± 0	1.1 ± 0.02	0.79 ± 0.09	0.4 ± 0.01	0.61 ± 0.01	0.32 ± 0.02
16:3ω3	0.15 ± 0.03	0.39 ± 0.06	0.11 ± 0.01	0.24 ± 0.02	0.37 ± 0.01	0.18 ± 0.02	0.47 ± 0.07
16:3ω4	0.23 ± 0.04	0.43 ± 0.02	0.41 ± 0.01	0.73 ± 0.03	0.22 ± 0.06	0.31 ± 0.02	0.83 ± 0.12
16:4ω1	tr.	0.11 ± 0.01	1.13 ± 0.04	0.7 ± 0.09	tr.	tr.	0.1 ± 0.01
16:4ω3	0.96 ± 0.08	0.9 ± 0.05	1.45 ± 0.06	0.9 ± 0.03	0.82 ± 0.05	1.19 ± 0.01	1.47 ± 0.09
18:2ω6	3.24 ± 0.22	3.37 ± 0.15	2.84 ± 0.09	3.05 ± 0.2	3.76 ± 0.22	4.05 ± 0.17	4.72 ± 0.39
18:2ω9	0.8 ± 0.06	1.05 ± 0.17	0.58 ± 0.06	1.02 ± 0.06	0.12 ± 0.01	0.15 ± 0.01	0.21 ± 0.01
18:3ω3	3.06 ± 0.29	3.64 ± 0.34	3.09 ± 0.12	1.95 ± 0.04	3.43 ± 0.15	3.94 ± 0.07	2.92 ± 0.1
18:3ω6	0.12 ± 0.02	0.2 ± 0.01	0.27 ± 0.01	0.22 ± 0.03	0.17 ± 0.01	0.2 ± 0.01	0.18 ± 0.02
18:4ω3	4.47 ± 0.41	4.13 ± 0.16	4.79 ± 0.1	2.43 ± 0.06	4.7 ± 0.29	5.5 ± 0.07	3.88 ± 0.24
20:4ω6	0.42 ± 0.04	0.5 ± 0.05	0.65 ± 0.01	0.71 ± 0.06	0.36 ± 0.05	0.32 ± 0.01	0.21 ± 0.01
20:5ω3	5.29 ± 0.58	5.34 ± 0.36	6.78 ± 0.13	3.17 ± 0.24	3.05 ± 0.37	4.46 ± 0.07	3.55 ± 0.37
22:6ω3	6.32 ± 0.81	5.76 ± 0.55	6.6 ± 0.3	2.28 ± 0.11	7.22 ± 1.07	6.84 ± 0.27	4.98 ± 0.64
**Σ PUFA**	**25.28 ± 2.39**	**26.06 ± 1.17**	**29.79 ± 0.27**	**18.2 ± 0.46**	**24.7 ± 2.03**	**28.25 ± 0.41**	**23.85 ± 1.27**
**ΣbrFA**	2.52 ± 0.15	3.4 ± 0.46	3.14 ± 0.12	4.47 ± 0.15	1.58 ± 0.07	1.9 ± 0.09	2.35 ± 0.1
**PUFA/SFA**	0.52 ± 0.07	0.67 ± 0.06	0.79 ± 0.01	0.48 ± 0.01	0.46 ± 0.06	0.57 ± 0.01	0.48 ± 0.03
**Σ EFA**	12.03 ± 1.42	11.61 ± 0.87	14.03 ±0.36	6.16 ± 0.32	10.63 ± 1.47	11.62 ± 0.33	8.74 ± 1

**16:1ω7/16:0**	0.24 ± 0.03	0.46 ± 0.03	0.66 ± 0.01	0.81 ± 0.04	0.19 ± 0.01	0.26 ± 0.01	0.3 ± 0.01
**EPA/DHA**	0.86 ± 0.04	0.95 ± 0.06	1.04 ± 0.05	1.41 ± 0.13	0.43 ± 0.01	0.66 ± 0.02	0.73 ± 0.03

Table S3: List and respective composition (% mean, SE, N=6) of trophic markers adopted in the present study in total particulate matter retrieved from shallow waters (8-20m) in the pristine (PS) and the anthropized sites (AS) over the 4 months (July, August, September, and October).

Fatty acid	Marker of	References	PS				AS		
			**Jul**	**Aug**	**Sept**	**Oct**	**Aug**	**Sept**	**Oct**
∑ai15:0, iso15:0, 18:1ω7	Bacteria	Meziane and Tsuchiya (2000; 2002)	7.31 (0.4)	11.02 (0.83)	8.49 (0.46)	13.00 (0.32)	4.16 (0.13)	5.75 (0.23)	4.83 (0.2)
∑ 14:0, 16:0, 18:0	Degraded organic matter	Bridier et al. (2021), Canuel and Zimmerman (1999), Connelly et al. 2015),	48.49 (3.51)	35.87 (2.1)	31.88 (0.48)	33.32 (0.41)	50.58 (2.87)	44.62 (0.82)	43.66 (0.50)
∑16:1ω7, 20:5ω3	Diatoms	Dalsgaard et al. (2003), Kelly and Scheibling (2012)	11.73 (0.86)	15.29 (0.6)	19.41 (0.17)	19.28 (0.79)	8.14 (0.30)	11.64 (0.32)	11.42 (0.07)
∑ 22:6ω3	Dinoflagellates	Kelly and Scheibling (2012)	6.32 (0.81)	5.76 (0.55)	6.6 (0.3)	2.28 (0.11)	6.97 (1.03)	4.98 (0.64)	6.84 (0.27)
∑18:2ω6, 18:3ω6, 18:4ω3	Green macroalgae	Bridier et al. (2021), Gaillard et al. (2017), Kelly and Scheibling, (2012)	7.83 (0.61)	7.7 (0.25)	7.9 (0.09)	5.7 (0.17)	8.42 (0.39)	8.78 (0.3)	9.74 (0.17)
∑ 18:1ω9	Brown macroalgae	Kelly and Scheibling (2012)	7.87 (0.43)	11.66 (0.32)	9.05 (0.37)	12.28 (1.35)	8.85 (0.84)	10.16 (0.56)	8.4 (0.2)

Table S4: Pairwise test results (two-way PERMANOVA) showing differences on species richness (S), Shannon-Weiner diversity (H’) and Pielou’s evenness (J’) among distances (D1, D2, D3), and months (July, August, September, and October) in the pristine (PS) and anthropized (AS) sites. Significant values (p<0.05) are indicated in bold.

**Richness (S)**							
	PS			*AS*			
Month	t-test	P(MC)	perms	t-test	P(MC)	perms	Distance
Jul, Aug	0.37	0.72	9392	**3.84**	**0.001**	**9738**	**D1, D2**
**Jul, Sept**	**3.28**	**0.004**	**6441**	**2.63**	**0.01**	**9729**	**D1, D3**
Jul, Oct	1.32	0.20	9700	1.04	0.31	6768	D2, D3
**Aug, Sept**	**2.60**	**0.02**	**9755**				
Aug, Oct	0.81	0.43	9731				
Sept, Oct	1.87	0.08	9725				

**Diversity (H’)**							
	PS			*AS*			
	t-test	P(MC)	perms	t-test	P(MC)	perms	
Month							*D1*
**Jul, Aug**	**3.54**	**0.002**	**9855**	**3.50**	**0.01**	**22**	**Jul, Aug**
**Jul, Sept**	**3.77**	**0.009**	**9853**	**10.69**	**0.0001**	**25**	**Jul, Sept**
**Jul, Oct**	**6.30**	**0.0001**	**9840**	0.61	0.56	25	Jul, Oct
Aug, Sept	0.08	0.93	9838	**5.76**	**0.001**	**35**	**Aug, Sept**
**Aug, Oct**	**3.99**	**0.0009**	**9845**	**3.95**	**0.01**	**35**	**Aug, Oct**
**Sept, Oct**	**4.43**	**0.0005**	**9825**	**16.92**	**0.0001**	**35**	**Sept, Oct**
Distance							*D2*
**D1, D2**	**3.36**	**0.003**	**9834**	1.67	0.14	35	Jul, Aug
**D1, D3**	**7.71**	**0.0001**	**9842**	**7.75**	**0.0001**	**32**	**Jul, Sept**
D2, D3	1.61	0.12	9819	0.24	0.81	31	Jul, Oct
				**4.53**	**0.005**	**32**	**Aug, Sept**
				1.91	0.11	34	Aug, Oct
				**13.18**	**0.0001**	**35**	**Sept, Oct**
							D3
				1.50	0.18	32	Jul, Aug
				1.62	0.16	35	Jul, Sept
				**2.92**	**0.03**	**35**	**Jul, Oct**
				0.43	0.68	34	Aug, Sept
				**18.73**	**0.0001**	**35**	**Aug, Oct**
				**25.65**	**0.0001**	**34**	**Sept, Oct**
							Jul
				1.19	0.27	24	D1, D2
				2.26	0.06	22	D1, D3
				**2.71**	**0.03**	35	**D2, D3**
							Aug
				1.91	0.10	35	D1, D2
				**6.54**	**0.0011**	35	**D1, D3**
				**7.10**	**0.0005**	32	**D2, D3**

							Sept
				**3.30**	**0.018**	**35**	**D1, D2**
				2.10	0.08	32	D1, D3
				**5.72**	**0.002**	**35**	**D2, D3**
							Oct
				**4.23**	**0.007**	**34**	**D1, D2**
				**4.64**	**0.0024**	**30**	**D1, D3**
				0.85	0.43	33	D2, D3

**Evenness (J')**							
	PS				*AS*		
	t-test	P(MC)	perms	t-test	P(MC)	perms	
Month							*D1*
**Jul, Aug**	**4.02**	**0.001**	**9817**	**3.23**	**0.01**	**35**	**Jul, Aug**
Jul, Sept	1.85	0.08	9814	**6.39**	**0.0006**	**35**	**Jul, Sept**
**Jul, Oct**	**6.92**	**0.0001**	**9824**	0.12	0.92	34	Jul, Oct
**Aug, Sept**	**2.63**	**0.01**	**9831**	**4.30**	**0.005**	**35**	**Aug, Sept**
**Aug, Oct**	**3.90**	**0.002**	**9844**	**3.38**	**0.02**	**35**	**Aug, Oct**
**Sept, Oct**	**6.29**	**0.0001**	**9846**	**6.74**	**0.001**	**35**	**Sept, Oct**
Distance							*D2*
**D1, D2**	**3.42**	**0.003**	**9827**	**2.69**	**0.04**	**35**	**Jul, Aug**
**D1, D3**	**7.99**	**0.0001**	**9799**	**8.36**	**0.0003**	**35**	**Jul, Sept**
**D2, D3**	**2.73**	**0.01**	**9833**	0.28	0.79	35	Jul, Oct
				**5.32**	**0.002**	**35**	**Aug, Sept**
				2.19	0.07	34	Aug, Oct
				**5.99**	**0.001**	**35**	**Sept, Oct**
							D3
				2.07	0.08	32	Jul, Aug
				2.17	0.07	35	Jul, Sept
				**3.36**	**0.01**	**34**	**Jul, Oct**
				0.31	0.76	35	Aug, Sept
				**14.74**	**0.0002**	**35**	**Aug, Oct**
				**15.23**	**0.0001**	**34**	**Sept, Oct**
							Jul
				0.52	0.62	35	D1, D2
				**3.13**	**0.02**	**35**	**D1, D3**
				**3.04**	**0.02**	**35**	**D2, D3**
							Aug
				0.65	0.53	35	D1, D2
				**8.20**	**0.0004**	**35**	**D1, D3**
				**8.37**	**0.0001**	**35**	**D2, D3**
							Sept
				0.14	0.94	35	D1, D2
				2.06	0.08	35	D1, D3
				**2.85**	**0.03**	**35**	**D2, D3**
							Oct
				0.07	0.95	35	D1, D2
				0.03	0.98	35	D1, D3
				0.05	0.96	35	D2, D3

Table S5: Pairwise results of two-way PERMANOVA showing differences in community composition among distances (D1, D2, D3) and months (July, August, September, and October) in the pristine (PS) and anthropized (AS) sites. Significant values ($p<0.05$) are indicated in bold.

		PS		AS	
Factor Distance	Pair-wise tests	t-test	P(MC)	t-test	P(MC)
July					
	D1, D2	2.83	**0.02**	2.77	**0.02**
	D1, D3	2.64	**0.03**	4.82	**0.001**
	D2, D3	1.10	0.32	2.68	**0.02**
August					
	D1, D2	4.64	**<0.001**	1.38	0.202
	D1, D3	4.22	**0.001**	9.19	**<0.001**
	D2, D3	2.28	**0.01**	3.83	**0.005**
September	D1, D2	1.26	0.237	6.80	**<0.001**
	D1, D3	3.23	**0.004**	7.99	**<0.001**
	D2, D3	3.56	**0.002**	4.72	**0.001**
October	D1, D2	2.34	**0.02**	4.40	**<0.001**
	D1, D3	3.54	**0.002**	4.52	**0.001**
	D2, D3	2.55	**0.01**	2.25	**0.03**
Factor Month	Pair-wise tests	t-test	P(MC)	t-test	P(MC)
D1					
	Jul, Aug	9.71	**<0.001**	1.01	0.35
	Jul, Sept	8.23	**<0.001**	4.98	**<0.001**
	Jul, Oct	7.50	**<0.001**	7.19	**<0.001**
	Aug, Sept	1.77	0.074	6.67	**<0.001**
	Aug, Oct	4.41	**<0.001**	11.69	**<0.001**
	Sept, Oct	3.61	**0.002**	4.12	**<0.001**
D2					
	Jul, Aug	7.85	**<0.001**	3.14	**0.001**
	Jul, Sept	8.05	**<0.001**	4.24	**0.003**
	Jul, Oct	9.23	**<0.001**	9.20	**<0.001**
	Aug, Sept	5.75	**<0.001**	1.61	0.12
	Aug, Oct	6.53	**<0.001**	8.15	**<0.001**
	Sept, Oct	4.76	**<0.001**	13.85	**<0.001**
D3					
	Jul, Aug	7.69	**<0.001**	5.62	**0.002**
	Jul, Sept	6.28	**<0.001**	5.46	**0.002**
	Jul, Oct	6.22	**<0.001**	8.88	**<0.001**
	Aug, Sept	1.77	0.08	2.11	0.05
	Aug, Oct	3.68	**<0.001**	9.91	**<0.001**
	Sept, Oct	3.61	**<0.001**	8.48	**<0.001**

Table S6: Pair-wise test results of the interaction of distance and month (two-way PERMANOVA) showing differences in the early recruitment of bivalves (*Hiatella arctica*, Mytilidade) and gastropods (*Lacuna* sp., and *Skenea* sp) among distances (D1, D2, D3), and months (July, August, September, and October) in the pristine (PS) and anthropized (AS) sites. Significant values (p<0.05) are indicated in bold.

			H. arctica		Mytilidae		*Skenea* sp.	
PS	*Factor Distance*	Pair-wise tests	t-test	P(MC)	t-test	P(MC)	t-test	P(MC)
	Jul	D1, D2	2.96	**0.02**	2.00	0.08	2.766	**0.03**
		D1, D3	2.33	0.05	5.19	**0.001**	2.689	**0.03**
		D2, D3	1.00	0.35	2.94	**0.01**	0.396	0.73
	Aug	D1, D2	4.38	**0.002**	5.77	**<0.001**	0.756	0.49
		D1, D3	1.90	0.10	7.70	**<0.001**	2.604	**0.01**
		D2, D3	2.23	0.05	2.96	**0.02**	2.749	**0.01**
	Sept	D1, D2	2.22	0.05	1.23	0.26	0.385	0.76
		D1, D3	1.16	0.25	6.06	**<0.001**	1.424	0.18
		D2, D3	0.53	0.68	8.69	**<0.001**	1.401	0.20
	Oct	D1, D2	0.47	0.73	2.64	**0.03**	0.369	0.79
		D1, D3	0.28	0.89	5.50	**<0.001**	2.897	**0.02**
		D2, D3	0.74	0.55	5.97	**<0.001**	2.319	**0.04**
	Factor Month	Pair-wise tests	t-test	P(MC)	t-test	P(MC)		
	D1	Jul, Aug	8.54	**<0.001**	1.54	0.17	6.24	**<0.001**
		Jul, Sept	8.40	**<0.001**	1.36	0.22	3.98	**0.001**
		Jul, Oct	6.09	**<0.001**	3.95	**0.002**	9.32	**<0.001**
		Aug, Sept	1.70	0.12	0.32	0.78	1.06	0.33
		Aug, Oct	3.59	**0.003**	4.78	**0.002**	0.87	0.42
		Sept, Oct	2.64	**0.023**	3.79	**0.005**	1.87	0.11
	D2	Jul, Aug	9.65	**<0.001**	0.60	0.61	2.19	**0.04**
		Jul, Sept	8.76	**<0.001**	4.72	**<0.001**	1.78	0.10
		Jul, Oct	8.45	**<0.001**	5.38	**<0.001**	3.23	**0.01**
		Aug, Sept	5.92	**<0.001**	6.52	**<0.001**	0.76	0.49
		Aug, Oct	1.93	**0.098**	7.51	**<0.001**	1.07	0.32
		Sept, Oct	6.23	**<0.001**	2.32	0.06	1.96	0.08
	D3	Jul, Aug	8.11	**<0.001**	1.23	0.27	3.78	**0.001**
		Jul, Sept	5.14	**<0.001**	2.73	**0.03**	1.18	0.29
		Jul, Oct	5.01	**<0.001**	6.63	**<0.001**	6.27	**<0.001**
		Aug, Sept	1.28	0.23	1.02	0.35	3.66	**0.001**
		Aug, Oct	2.35	**0.03**	4.24	**0.002**	1.38	0.2
		Sept, Oct	2.59	**0.01**	3.84	**0.005**	6.03	**<0.001**

			H.arctica		Mytilidae		*Lacuna* sp.		*Skenea* sp.	
AS	*Factor Distance*	Pair-wise tests	t-test	P(MC)	t-test	P(MC)	t-test	P(MC)	t-test	P(MC)
	Jul	D1, D2	2.81	**0.02**	1.22	0.27	2.82	**0.02**	3.10	**0.01**
		D1, D3	4.87	**<0.001**	8.77	**0.001**	4.28	**0.002**	0.85	0.45
		D2, D3	2.64	**0.03**	3.67	**0.01**	3.20	**0.01**	2.05	0.06
	Aug	D1, D2	1.29	0.25	2.46	**0.03**	2.14	0.0	1.72	0.08
		D1, D3	9.68	**<0.001**	4.34	**0.002**	11.99	**<0.001**	0.43	0.83
		D2, D3	3.61	**0.006**	2.68	**0.03**	8.67	**<0.001**	1.26	0.23
	Sept	D1, D2	5.57	**<0.001**	3.22	**0.02**	7.03	**<0.001**	1.12	0.31
		D1, D3	6.20	**<0.001**	3.87	**0.01**	9.27	**<0.001**	1.31	0.19
		D2, D3	4.88	**0.002**	2.29	0.06	3.73	**0.008**	0.76	0.59
	Oct	D1, D2	7.83	**<0.001**	2.02	0.086	0.19	0.90	0.96	0.41
		D1, D3	5.47	**<0.001**	6.88	**<0.001**	0.79	0.46	1.67	0.07
		D2, D3	1.92	0.09	5.59	**0.002**	0.59	0.58	3.62	**0.002**
	Factor Month	Pair-wise tests	t-test	P(MC)	t-test	P(MC)	t-test	P(MC)	t-test	P(MC)
	D1	Jul, Aug	0.75	0.49	4.13	**0.003**	3.71	**0.01**	3.20	**0.003**
		Jul, Sept	4.09	**0.01**	7.24	**<0.001**	10.12	**<0.001**	3.60	**0.001**
		Jul, Oct	6.95	**<0.001**	10.61	**<0.001**	6.39	**<0.001**	3.11	**0.003**
		Aug, Sept	5.00	**<0.001**	1.39	0.21	13.69	**<0.001**	0.74	0.62
		Aug, Oct	10.32	**<0.001**	0.80	0.46	5.22	**0.002**	0.87	0.51
		Sept, Oct	4.58	**<0.001**	1.47	0.19	4.35	**0.003**	0.58	0.74
	D2	Jul, Aug	3.02	**0.01**	5.47	**0.001**	1.29	0.24	5.36	**<0.001**
		Jul, Sept	3.99	**0.004**	7.71	**<0.001**	17.36	**<0.001**	3.54	**0.001**
		Jul, Oct	9.93	**<0.001**	5.44	**<0.001**	6.13	**<0.001**	8.12	**<0.001**
		Aug, Sept	0.28	0.83	1.11	0.30	11.45	**<0.001**	0.92	0.44
		Aug, Oct	9.03	**<0.001**	2.35	**0.05**	5.59	**<0.001**	2.77	**0.01**
		Sept, Oct	18.79	**<0.001**	7.46	**<0.001**	8.42	**<0.001**	1.87	0.07
	D3	Jul, Aug	5.74	**<0.001**	8.63	**<0.001**	2.36	**0.05**	1.94	**0.03**
		Jul, Sept	5.37	**0.001**	8.67	**<0.001**	5.51	**<0.001**	2.57	**0.01**
		Jul, Oct	7.14	**<0.001**	8.70	**<0.001**	0.73	0.51	3.21	**0.003**
		Aug, Sept	1.14	0.29	0.49	0.66	8.98	**<0.001**	1.00	0.39
		Aug, Oct	7.46	**<0.001**	0.74	0.50	3.88	**0.01**	1.14	0.30
		Sept, Oct	6.83	**<0.001**	0.25	0.84	11.79	**<0.001**	0.48	0.82

Note: Due to the lack of interaction between distance and month factors on *Lacuna* sp. abundances at the PS site, the pairwise tests were not included in this table but are presented in the results section in the main document.

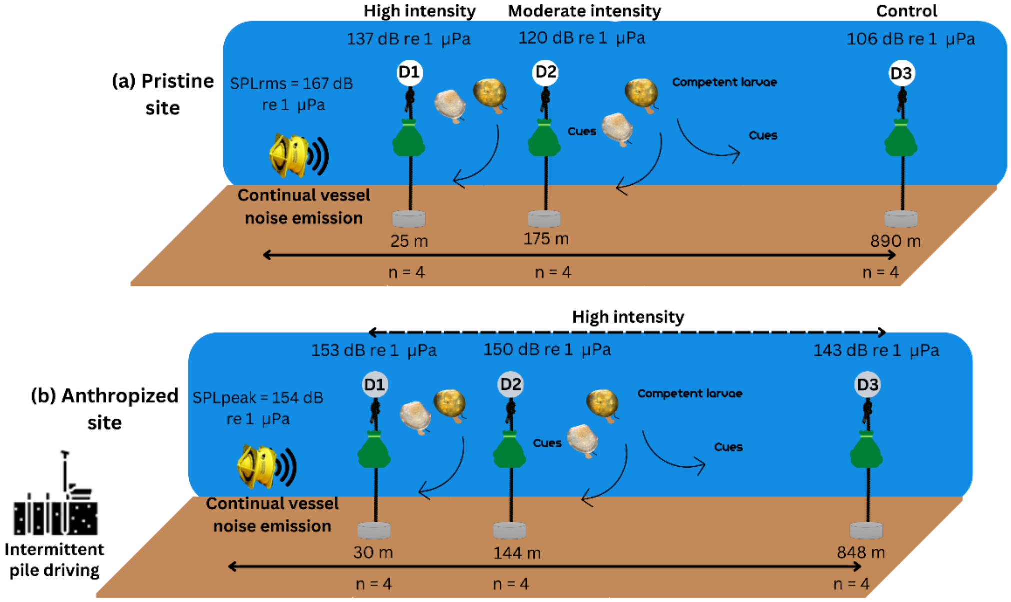

Figure S1: Graphical representation of the experimental design carried out at the pristine (a) and anthropized sites (b). Recruits (pediveligers, post-larvae and juveniles) of benthic invertebrates were exposed to a gradient of vessel noise (D1 = 137 dB at 25m, D2 = 120 dB at 175m and D3= 106 dB re 1 µPa at 890m) or a mix of vessel noise and pile driving noises (D1 = 153 dB, D2 = 150 dB and D3 = 143 dB re 1 µPa) along transects, where artificial collectors were placed at each station (n = 4). Illustration created on Canva using icons from Canva and Icon8, and photographs taken by the author.

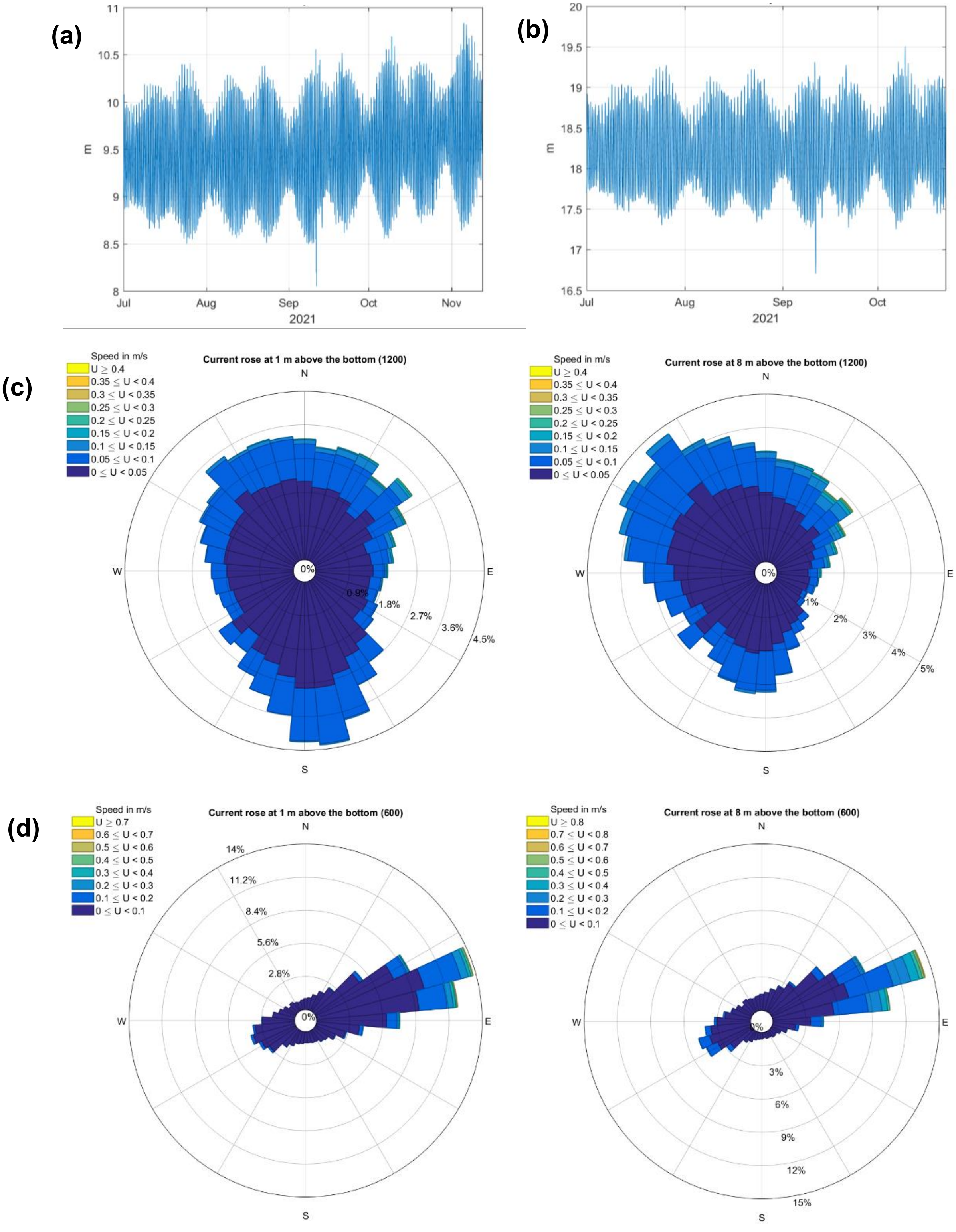

Figure S2: Characterization of tides and currents in both pristine (PS, **a, c**) and the anthropized (AS, **b, d**) experimental sites during the sampling months of July, August, September, and October.

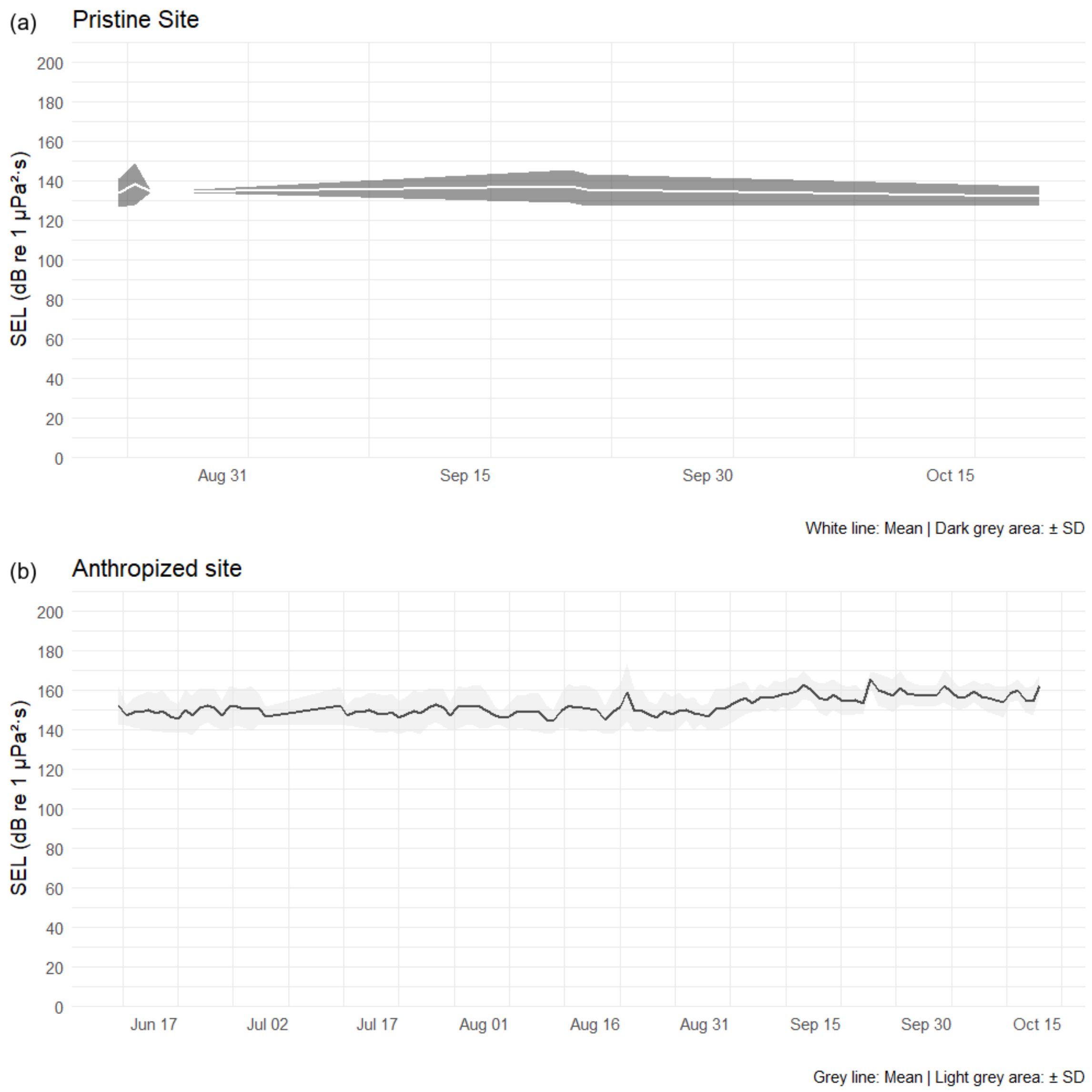

Figure S3: Acoustic characterization at pristine (a) and anthropized (b) sites with daily mean (±SD) of sound exposure level (SEL1min, dB re 1 µPa^{2}s).

References

Bligh, E.G. and Dyer, W.J. 1959. “A rapid method of total lipid extraction and purification” *Canadian Journal of Biochemistry and Physiology* 37 (8): 911-917.

Bridier, Guillaume, Tarik Meziane, Jacques Grall, Laurent Chauvaud, Sébastien Donnet, Pascal Lazure, and Frédéric Olivier. 2021. “Sources, Quality and Transfers of Organic Matter in a Highly-Stratified Sub-Arctic Coastal System (Saint-Pierre-et-Miquelon, NW Atlantic).” *Progress in Oceanography* 190 (November 2020). https://doi.org/10.1016/j.pocean.2020.102483.

Cannuel, Elizabeth A., and Andrew R. Zimmerman. 1999. “Composition of Particulate Organic Matter in the Southern Chesapeake Bay: Sources and Reactivity.” *Estuaries* 22 (4): 980–94. https://doi.org/10.2307/1353077

Chynel, Mathias, Sofia Rockomanovic, Gwenael Abril, Glenda Barroso, Humberto Marotta, Wislon Machado, Christian J. Sanders, Najet Thiney, and Tarik Meziane. 2022. “Contrasting Organic Matter Composition in Pristine and Eutrophicated Mangroves Revealed by Fatty Acids and Stable Isotopes (Rio de Janeiro, Brazil).” *Estuarine, Coastal and Shelf Science* 277 (June). https://doi.org/10.1016/j.ecss.2022.108061

Connelly, Tara L., James W. McClelland, Byron C. Crump, Colleen T. E. Kellogg, and Kenneth H. Dunton. 2015. “Seasonal Changes in Quantity and Composition of Suspended Particulate Organic Matter in Lagoons of the Alaskan Beaufort Sea.” *Marine Ecology Progress Series* 527: 31–45. https://doi.org/10.3354/meps11207

Dalsgaard, Johanne, Michael St. John, Gerhard Kattner, Dorthe Müller-Navarra, and Wilhelm Hagen. 2003. “Fatty Acid Trophic Markers in Pelagic Marine Environment”. *Advances in Marine Biology* 46: 225-332.

Gaillard, Blandine, Tarik Meziane, Réjean Tremblay, Philippe Archambault, Martin E. Blicher, Laurent Chauvaud, Soren Rysgaard, and Frédéeric Olivier. 2017. “Food Resources of the Bivalve Astarte Elliptica in a Sub-Arctic Fjord: A Multi-Biomarker Approach.” *Marine Ecology Progress Series* 567 (Wassmann 2011): 139–56. https://doi.org/10.3354/meps12036

Graeve, Martin , Gerhard Kattner, Christian Wiencke and Ulf Karsten. 2002. Fatty acid composition of Arctic and Antarctic macroalgae:indicators for phylogenetic and trophic relationships , *Marine Ecology-Progress Series*, 231 , pp. 67-74 . https://doi.org/10.3354/meps231067

Kelly, Jennifer R., and Robert E. Scheibling. 2012. "Fatty Acids as Dietary Tracers in Benthic Food Webs." *Marine Ecology Progress Series* 446: 1–22. https://doi.org/10.3354/meps09559

Leal, Inês, Réjean Tremblay, and Augusto A. V. Flores. 2022. "Allochthonous Subsidies Drive Early Recruitment of a Subtropical Foundation Species." *Oikos* 2022 (7): 1–16. https://doi.org/10.1111/oik.08991

Meziane, T., S. Y. Lee, P. L. Mfilinge, P. K.S. Shin, M. H.W. Lam, and M. Tsuchiya. 2007. "Inter-Specific and Geographical Variations in the Fatty Acid Composition of Mangrove Leaves: Implications for Using Fatty Acids as a Taxonomic Tool and Tracers of Organic Matter." *Marine Biology* 150 (6): 1103–13. https://doi.org/10.1007/s00227-006-0424-z

Meziane, Tarik, and Makoto Tsuchiya. 2002. "Organic Matter in a Subtropical Mangrove-Estuary Subjected to Wastewater Discharge: Origin and Utilisation by Two Macrozoobenthic Species." *Journal of Sea Research* 47 (1): 1–11. https://doi.org/10.1016/S1385-1101(01)00092-2

Meziane, Tarik, and Makoto Tsuchiya. 2000. "Fatty Acids as Tracers of Organic Matter in the Sediment and Food Web of a Mangrove/Intertidal Flat Ecosystem, Okinawa, Japan." *Marine Ecology Progress Series* 200 (Motonaga 1977): 49–57. https://doi.org/10.3354/meps200049
